# Nonthermal excitation effects mediated by sub-terahertz radiation on hydrogen exchange in ubiquitin

**DOI:** 10.1016/j.bpj.2021.04.013

**Published:** 2021-05-01

**Authors:** Yuji Tokunaga, Masahito Tanaka, Hitoshi Iida, Moto Kinoshita, Yuya Tojima, Koh Takeuchi, Masahiko Imashimizu

**Affiliations:** 1 Cellular and Molecular Biotechnology Research Institute, National Institute of Advanced Industrial Science and Technology, Tokyo, Japan; 2 Research Institute for Measurement and Analytical Instrumentation, National Institute of Advanced Industrial Science and Technology, Tsukuba, Japan; 3 Research Institute for Physical Measurement, National Institute of Advanced Industrial Science and Technology, Tsukuba, Japan

## Abstract

Water dynamics in the hydration layers of biomolecules play crucial roles in a wide range of biological functions. A hydrated protein contains multiple components of diffusional and vibrational dynamics of water and protein, which may be coupled at ∼0.1-THz frequency (10-ps timescale) at room temperature. However, the microscopic description of biomolecular functions based on various modes of protein-water-coupled motions remains elusive. A novel approach for perturbing the hydration dynamics in the subterahertz frequency range and probing them at the atomic level is therefore warranted. In this study, we investigated the effect of klystron-based, intense 0.1-THz excitation on the slow dynamics of ubiquitin using NMR-based measurements of hydrogen-deuterium exchange. We demonstrated that the subterahertz irradiation accelerated the hydrogen-deuterium exchange of the amides located in the interior of the protein and hydrophobic surfaces while decelerating this exchange in the amides located in the surface loop and short 3_10_ helix regions. This subterahertz-radiation-induced effect was qualitatively contradictory to the increased-temperature-induced effect. Our results suggest that the heterogeneous water dynamics occurring at the protein-water interface include components that are nonthermally excited by the subterahertz radiation. Such subterahertz-excited components may be linked to the slow function-related dynamics of the protein.

## Significance

It has been shown that at physiological temperatures in aqueous solution, fluctuating dynamics of protein and coupled water molecules occur in the subterahertz frequency range. If so, does the externally applied alternating electromagnetic field with subterahertz frequency resonantly interact with the protein-water-coupled dynamics and nonthermally influence protein functions? Can we use subterahertz radiation energy as an efficient tool for understanding the microscopic details of elementary processes for biomolecular functions, including the contribution of water? We challenge this open question, combining the 0.1-THz irradiation with NMR-based measurements at the atomic level. In particular, we demonstrate that the applied subterahertz radiation energy leads to a solvent effect on structural dynamic changes of ubiquitin in a manner that cannot be explained by temperature increase.

## Introduction

The hydration layers surrounding proteins play crucial roles in biological functions such as folding, enzymatic reactions, and protein-protein interactions (1, 2, 3). The basic mechanisms underlying water dynamics in the hydration layers of biomolecules have been extensively investigated using spectroscopic approaches and molecular dynamics simulations (4,5). These studies have revealed that diffusive protein and hydration dynamics occur on the same timescale on the order of 10 ps at physiological temperatures (6,7). Because protein and hydration dynamics show similar temperature dependency, a mechanism of coupled protein-hydration water relaxation has been suggested (6,7). On the same or slightly faster timescale, the low-frequency vibrational mode of proteins also overlaps with the diffusive protein and hydration dynamics (8,9). Such a variety of diffusional and vibrational modes of protein and water dynamics at this timescale would be directly linked to elementary molecular processes involved in the expression of biological functions. However, these dynamics show similar dielectric responses, making it difficult to derive a simplified description of biomolecular functions based on microscopic hydration properties (10,11). Indeed, such a direct link between the fast dynamics and biological functions is not supported by a recent study of NMR relaxation (12).

The dynamics with a 10-ps timescale correspond to a frequency of 0.1 THz in an oscillation period. Oscillating protein motions of 0.1-THz resonant frequency were first described by normal mode analysis over 30 years ago (13). In an actual aqueous protein solution, water’s rotational relaxations, rather than protein motions, are detected as the dominant dielectric responses at ∼0.1 THz (8,14, 15, 16, 17). However, as mentioned above, there are multiple superimposed components associated with protein and water motions in this frequency region at physiological temperatures. This prompted us to hypothesize that the 0.1-THz radiation may directly interact with biologically relevant protein-water-coupled motions observed at the same subterahertz frequency. More precisely, the subterahertz excitation energy is possibly redistributed to the hydrogen-bond network specific to the protein-water interface before thermal dissipation to the bulk water, thereby altering hydration dynamics. Notably, protein and hydration dynamics influence each other (8,10,11) and are strongly dependent on temperature (8,11). Therefore, subterahertz and thermal excitations might have nearly the same consequence unless the subterahertz excitation energy remains localized in the hydration dynamics largely beyond 10 ps. In other words, the subterahertz excitation energy must be adiabatically retained in the specific hydrogen-bond network localized and restrained at the protein-water interface until the timescales relevant to the structural changes of proteins.

Innovative approaches for analyzing biomacromolecular dynamics such as the optical Kerr effect, extraordinary acoustic Raman, and kinetic and anisotropic terahertz spectroscopies have been developed (9,18, 19, 20, 21). Notably, these approaches revealed that collective water and biomolecule (protein or DNA) dynamics could persist well beyond the 10-ps timescale at physiological temperature, contributing to enzymatic activities on biological timescales (22, 23, 24). In addition, several studies using different (sub)terahertz light sources have shown that (sub)terahertz excitation alters the kinetics of DNA conformations and assemblies that are slow enough to be detected by conventional biochemical techniques (25). Moreover, it changes the electron density in the hydrated lysozyme crystal (26) and significantly affects actin polymerization (27) and gene expression (28) under physiological conditions. The above studies have performed well-designed control experiments that excluded the possible effect of (sub)terahertz-radiation-induced temperature increase. Thus, we can assume that the effect of subterahertz excitation on protein and hydration dynamics can be discriminated from the heating effect, and the difference would be reflected in the slow relaxation processes detectable by proton exchange reactions occurring on timescales as slow as minutes to hours at room temperature (29,30). The consequence of subterahertz excitation in the slow reactions occurring in an aqueous solution has not yet been directly assessed at the atomic level.

To monitor such slow relaxation processes after subterahertz or thermal excitation, we developed a novel, to our knowledge, method that combines a klystron-based 0.1-THz irradiation system (subterahertz-klystron) with NMR-based hydrogen-deuterium exchange (HDX), which we have termed THz-HDX. NMR-based HDX is a well-established technique for analyzing protein dynamics in aqueous systems (31,32). Herein, we used ubiquitin (Ub) as a model protein. Ub is a small, 76-amino acid globular protein found ubiquitously in all eukaryotic organisms (30,33), and it is extremely stable under heat denaturation and exhibits slow amide proton exchange kinetics (34,35). In addition, Ub has a tightly folded *β*-sheet, an *α*-helix, a short 3_10_-helix, and surface loops between these elements in its compact structure, allowing physicochemical study of protein structure with high generality. Therefore, Ub has been intensively investigated for assessing conformational and hydrational changes upon external perturbations using NMR-based HDX and relaxation techniques (29,30,36, 37, 38, 39), X-ray crystallography coupled with molecular dynamics simulations (40, 41, 42), and terahertz spectroscopy (43,44).

Using the THz-HDX method, we characterized the amide proton exchange kinetics of Ub after subterahertz irradiation and compared them with those after temperature increase mediated by heat conduction. We demonstrated that these two perturbations differently influenced the HDX in Ub. The subterahertz irradiation primarily increased the HDX of amides clustered in an interior region and a hydrophobic surface but decreased the amide HDX in the surface loop and short 3_10_ helix regions. We also found that such subterahertz-induced effects were qualitatively opposite to those induced by increased temperature. This result is consistent with the view that heterogeneous water and protein dynamics at the interface are nonthermally excited by subterahertz irradiation.

## Materials and methods

### Subterahertz source

**Figure 1:**
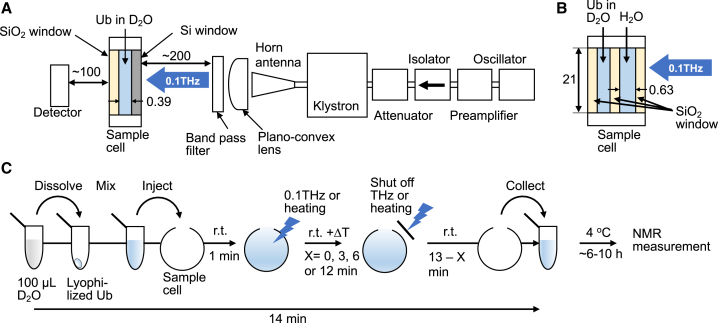
Schematic representation of subterahertz radiation experimental setup. (*A* and *B*) Subterahertz irradiation (*A*) and subterahertz-dependent heat conduction (*B*) to ubiquitin (Ub) solution using the same klystron-based subterahertz source. Devices for subterahertz radiation were connected through rectangular waveguides, WR-10. The length unit is shown in millimeter. See Materials and methods for details. (*C*) THz-HDX experiment. Lyophilized Ub was dissolved in 100 *μ*L D_2_O to obtain 0.5 mM Ub solution, which was thoroughly mixed using a vortex mixer and was then injected into the sample cell within 1 min at room temperature (rt) of ∼25°C. The sample was incubated for 13 min at rt and was subjected to temperature increase (+ΔT) caused by subterahertz radiation or heat conduction for a variable time (X = 0, 3, 6, or 12 min). The experiment conducted at X = 0 corresponds to control. After 14 min of dissolution in D_2_O, Ub solution was collected in the sample tube and stored at 4°C until NMR-based HDX measurement was performed. The time interval between the sample collection and NMR measurement per condition is shown in Fig. S2.

Subterahertz irradiation experiments were performed using a klystron-based subterahertz source (45). A schematic representation of the experimental setup is illustrated in Fig. 1 *A*. The terahertz source comprised a W-band oscillator, a preamplifier, an isolator, a direct reading attenuator (CAR-1050-01; WiseWave, Torrance, CA), a klystron (Extended Interaction Klystron VKB2461; CPI, Palo Alto, CA), and a pyramidal horn antenna. All devices were connected through rectangular waveguides, WR-10. This source can generate 95 ± 0.25 GHz of radiation, a 10-kHz repetition rate, and a 0.8-*μ*s pulse width of a square wave. The emitted subterahertz radiation is roughly collimated by a quartz plano-convex lens and monochromated by a bandpass filter with a center frequency of 95 GHz (MMBPF40; Joint Technology Development Platform, Kyoto, Japan). The subterahertz radiation diameter was estimated to be ∼20 mm at the sample position by measuring the full width at half maximum. The sample solution was set at a distance of 200 mm from the bandpass filter (Fig. 1 *A*). The maximal power density of the 0.1-THz radiation that was irradiated on the sample with an effective diameter of 21 mm was estimated to be 250 mW/cm^2^ using a sensitive thermal sensor (3A-P-THz; Ophir Optronics Solutions, Jerusalem, Israel), which is specific for terahertz and subterahertz radiation. The efficiency of 0.1-THz radiation was determined by comparing the radiation powers measured using a reference calorimeter (46) and the detector. The attenuator controls the power density. The subterahertz power was monitored using the detector set downstream of the sample cell during the irradiation experiment.

### Subterahertz irradiation and heat treatment of Ub

Lyophilized Ub was dissolved in 100 *μ*L of D_2_O, thoroughly mixed using a vortex mixer, and then injected into a demountable liquid transmission cell (DLC-M25; Harrick Scientific Products, Ossining, NY) within 1 min at room temperature (∼25°C; Fig. 1 *C*). The pathlength of the D_2_O sample containing Ub was adjusted to 390 *μ*m using a Teflon spacer. A high-resistivity float zone silicon window (Tydex, St. Petersburg, Russia) and SiO_2_ window (QPSQ-25C02-10-5; SIGMAKOKI, Tokyo, Japan), each with a thickness of 2.0 mm and an effective diameter of 21 mm, were placed upstream and downstream of the sample cell, respectively (Fig. 1, *A* and *B*). The conditions of the sample solution were visually identified through the SiO_2_ window. Approximately 40% of the radiation was lost owing to reflection on the silicon window; therefore, the remaining 60% of the subterahertz radiation was transmitted to the aqueous sample. Consequently, the low- and high-power density of subterahertz radiation transmitted to the sample surface was estimated to be 18 and 90 mW/cm^2^, respectively, each of which was used as the subterahertz exposure condition. Owing to the large absorption coefficient of water, 97% of the transmitted radiation was absorbed by the 390-*μ*m-thick sample solution (*α* = 83 cm^−1^ at 25°C, 0.1 THz) (45). The Ub sample was exposed to either subterahertz radiation or heating (terahertz radiation-derived heat conduction) using the same klystron-based 0.1-THz source under ambient conditions (∼25°C) (Fig. 1, *A* and *B*). For heating, the subterahertz wave was irradiated onto 630-*μ*m-thick water (∼220 *μ*L) inserted between two SiO_2_ windows (Fig. 1 *B*). The temperature of the sample was measured using a K-type thermocouple.

### Preparation of isotope-labeled yeast Ub

Uniformly ^13^C/^15^N-labeled recombinant yeast Ub was prepared as previously described (47). Briefly, *Escherichia coli* BL21(DE3) cells were transformed with the plasmid vector pET-26b (Merck Millipore, Burlington, MA), harboring the gene encoding Ub (76 amino acids) at *Nde*I/*Sal*I restriction sites, without additional sequences. Five colonies of transformed cells cultured on a Luria-Bertani-agarose plate containing 50 *μ*g/mL kanamycin were inoculated into 10 mL of Luria-Bertani-kanamycin medium in a 50-mL conical tube and then incubated by shaking at 37°C for 6 h. Next, the cell pellet obtained after centrifugation was resuspended in 500 mL of M9-kanamycin medium in a 2-L Sakaguchi flask, containing [^15^N] ammonium chloride and [^13^C_6_] D-glucose as the sole nitrogen and carbon sources, respectively, and then incubated while shaking at 37°C. When the optical density at 600 nm reached 0.6–0.8, isopropyl-*β*-D-1-thiogalactopyranoside was supplemented at a concentration of 1 mM to induce the overexpression of Ub and further incubated for 6 h. Cells were harvested by centrifugation and stored at −80°C until purification. The cell pellet was resuspended in 30 mL lysis buffer containing 50 mM sodium acetate at pH 5.5. Cells were lysed by sonication on ice, and the cell debris was removed by centrifugation at 20,000 × *g* at 8°C for 1 h. The supernatant was then loaded onto an SP Sepharose Fast Flow column (Cytiva, Marlborough, MA) and washed with lysis buffer, and bound proteins were eluted using a 50–300 mM NaCl gradient. Ub was eluted into 100–150 mM NaCl fractions and further purified using size exclusion chromatography using a HiLoad 16/60 Superdex 75 prep grade column (Cytiva). The elution fraction was stored at −30°C until use in NMR experiments.

### THz-HDX experiments

A stock solution of Ub in 50 mM sodium phosphate buffer (pH 6.4; also see main text for other pH values tested) containing 50 mM NaCl was diluted to 0.2 mM with the same buffer. Then, 250 *μ*L of Ub solution was added to 1.5-mL tubes and flash frozen in liquid nitrogen, followed by lyophilization in a freeze-dryer FDU-2100 (EYELA, Tokyo, Japan) at room temperature and under a pressure of ∼5.5 Pa for 36 h. This treatment eliminates bulk water while preserving bound water on the protein surface (48,49).

The HDX experiment was initiated by dissolving lyophilized Ub in 100 *μ*L D_2_O to prepare a 0.5-mM Ub solution at room temperature (Fig. 1 *C*). The D_2_O solution of Ub was immediately transferred into the sample cell, and subterahertz irradiation was initiated 1 min after dissolution and was continued for 3, 6, or 12 min. After irradiation, the Ub solution was collected in a 1.5-mL tube and stored at 4°C to minimize subsequent HDX (Fig. 1 *C*). For all samples, the time from D_2_O addition at room temperature to storage at 4°C was fixed at 14 min. Temperature control (TC) experimental samples were prepared for each time point by inserting a water layer between the Ub solution and subterahertz source to shield the Ub solution from direct irradiation while allowing passive temperature rise via thermal conduction from the subterahertz-heated water (Fig. 1 *B*). In addition, general control (GC) experimental samples not subjected to subterahertz irradiation or temperature elevation but only incubated in the sample cell were prepared.

All samples were mixed with 150 *μ*L of ice-cold D_2_O-based buffer solution with the same composition as the ubiquitin stock buffer and then transferred into prechilled microtubes with an outer diameter of 5 mm (Shigemi, Tokyo, Japan) at 4°C. NMR experiments were performed using a Bruker Avance III 800 MHz spectrometer equipped with a TXO triple-resonance cryoprobe (Bruker, Billerica, MA). The samples were loaded onto a magnet set at 4°C under nitrogen gas flow. After frequency lock, tuning and matching of radiofrequency coils, and shimming, three ^1^H-^15^N SOFAST-HMQC spectra were sequentially acquired at 4°C (50). Each of the three spectra were acquired in 5 min 55 s, with acquisition times in the direct and indirect dimensions of 45.9 ms (*t*2, ^1^H) and 43.2 ms (*t*1, ^15^N), spectral widths of 14 and 32 ppm, number of scans of four per indirect *t*1 point, and with a interscan delay of 0.3 s. Three spectra were summed (∼18 min in total) to enhance S/N, as we verified the absence of progress of HDX during the measurement. We observed no significant differences in the average signal intensities among the three spectra (overall, the standard deviation was 2–3% of the mean). After the measurement, samples were incubated at room temperature for >12 h to equilibrate HDX, followed by a ^1^H-^13^C constant-time HSQC experiment (51).

All the experiments were repeated two or more times, and representative results are shown. The HDX and NMR experiments were performed at the AIST Tsukuba Central and AIST Tokyo Waterfront, respectively. All samples were kept at 4°C for ∼2 h of transportation between the two institutions.

### NMR spectroscopic data analyses

Time-domain data were multiplied by Gaussian and *π*/2-shifted squared sine window functions on *t*2 free induction decays and *t*1 interferograms, respectively, followed by Fourier transformation and phase and baseline corrections in the TopSpin 2.1 software (Bruker). The spectra were analyzed using Sparky (52). To account for the effects of subtle differences in Ub concentrations among samples, amide signal intensities were normalized using signals from unexchangeable groups as internal controls. For this purpose, the heights of 37 methyl resonances in the ^1^H-^13^C constant-time HSQC spectra were used (Fig. S1). The ratio of subterahertz intensity (or TC) to that of GC was used to evaluate the effects of subterahertz irradiation (or temperature elevation) on HDX.

(1) $$R_{t}=\frac{I_{i,THz\;\left(or\;TC\right),\;t}}{I_{i,\;GC,t}},$$

where *t* = 3, 6, and 12 min. Under subterahertz wave- or heat-induced HDX acceleration, this ratio is <1, whereas under deceleration, it is >1. However, because the temperature elevation induced by subterahertz irradiation equilibrates after ∼3 min, we used differences in *R*_*t*_ at 6 and 12 min from *R*_3 min_ as quantitative criteria for deciding whether the HDX is accelerated or decelerated.

(2) $$\Delta R=R_{t}-R_{3\;min}.$$

If HDX is accelerated (or decelerated) by subterahertz irradiation or heating after temperature equilibration, Δ*R* will be negative (or positive) to an extent larger than its error,

(3) $$\Delta R+Error\left(\Delta R\right)<\;0\to \text{acceleration,}$$

(4) $$\Delta R-Error\left(\Delta R\right)>\;0\to \text{deceleration.}$$

*Error*(Δ*R*) was defined using the sum of each *Error* at two time points *t* and 3 min as follows:

(5) Error(ΔR)=[Error(Rt)+Error(R3min)]2,

where each *Error*(*R*_*t*_) was estimated from the signal/noise ratio (SNR) considering error propagation according to (53):

(6) Error(Rt)=Rt(1SNRTHz(TC),t)2+(1SNRGC,t)2.

## Results

We monitored the main-chain amide protons of Ub as they were being exchanged with solvent deuterons after the dissolution of the lyophilized Ub in D_2_O. In general, lyophilization eliminates bulk H_2_O from protein solutions while preserving bound H_2_O on protein surfaces (48,49). Although lyophilization could induce protein unfolding or structural collapse (54), no such effect was observed in the Ub used in our experiment (Supporting materials and methods; Fig. S3). For subterahertz excitation, we applied intense 0.1-THz electromagnetic pulses with high (90 mW/cm^2^) or low (18 mW/cm^2^) power density to the Ub solution by constructing an optical setup using subterahertz klystron (Fig. 1 *A*). We measured the elevation in the volume-averaged temperature for the subterahertz-irradiated sample by immersing a thermocouple in the sample solution. The temperature gradually increased for 3 min, and the plateau was estimated to be 5 and 0.3°C for the high- and low-power density irradiations, respectively. For the TC experiment, we similarly raised the sample temperature by 5°C through a water layer attached to the subterahertz-exposed surface of the SiO_2_ window (Fig. 1 *B*). This layer completely absorbed the subterahertz radiation and allowed the heat transfer to the sample in a beam-power-controlled manner without direct subterahertz irradiation. After irradiation or heating for 3, 6, or 12 min, all samples were further incubated at room temperature to ensure a constant duration of 14 min after dissolving in D_2_O (Fig. 1 *C*). The Ub solution was then stored for 6–10 h at 4°C to minimize the subsequent exchange until NMR measurement. For the GC experiment, the same experiment using the same subterahertz-klystron setup was conducted by cutting off the terahertz radiation.

We obtained two-dimensional amide ^1^H-^15^N SOFAST-HMQC spectra of Ub after exposure to 90- or 18-mW/cm^2^ subterahertz radiation. In these spectra, resonances of nonproline amino acid residues were observed at distinct chemical shifts, whose intensities reflect the extent of HDX, as the deuterated amide moieties after HDX were not detected in the experiment. The spectra of the amino acid residues were compared with those of TC and GC. We verified that there was no significant difference in the spectral patterns with and without subterahertz irradiation, indicating that the applied intense 0.1-THz pulses did not cause irreversible changes (e.g., denaturation, oxidation, deamination, and isomerization) to the protein structure (Fig. S4). The target residues of interest were selected by excluding those with 1) fast HDX rates, that is, being exchanged during the sample storage time at 4°C within 6–10 h (Fig. S2); 2) relatively low signal intensity (signal/noise ratio of <33.3, which corresponds to >3% noise level relative to the intensity); and 3) overlapping of two signals.

For the selected 21 residues, we estimated the residue-specific HDX rate for the GC sample based on real-time NMR measurement, which showed that HDX rate of these residues are smaller than ∼0.1 h^−1^ (Fig. S5). It should be noted that the signal intensity of several residues was increased time dependently, supposedly because of reduced dipolar broadening (55). However, this should not substantially affect the interpretation of THz-HDX data, as the change would have been equilibrated within the delay of 6–10 h before NMR measurements (Fig. S2).

**Figure 2:**
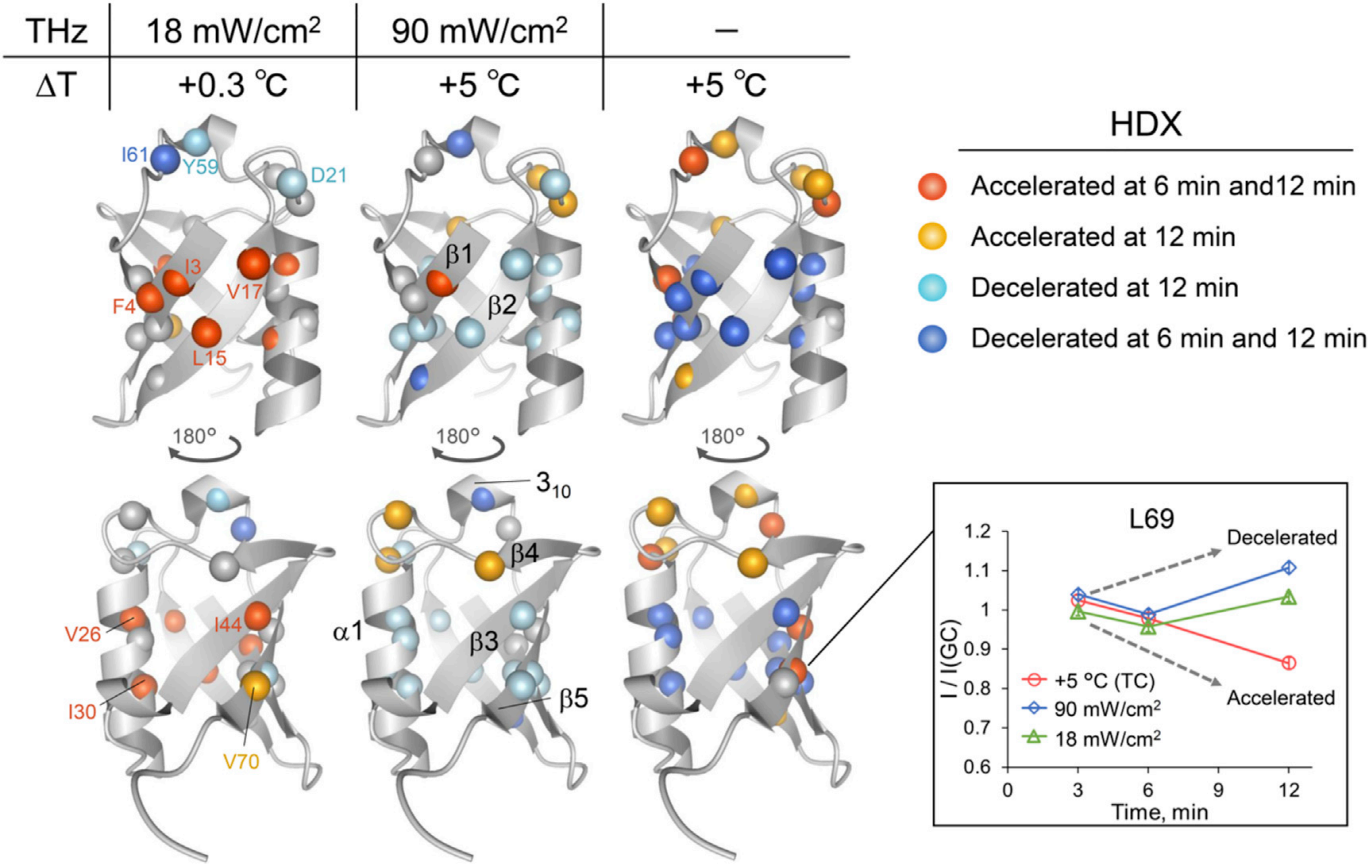
Effect of subterahertz irradiation at low (*left*) and high (*middle*) power density and temperature increase (*right*) on the amide proton exchange of Ub. The tertiary Ub structures with 180° rotation are shown (Protein Data Bank, PDB: 1UBQ) (42). Amide nitrogen atoms of the analyzed residues are shown (see main text for details). Amino acid residues were mapped in the Ub structure when the HDX of the main chain amide groups was accelerated or decelerated. An example of L69 is shown in an inset. The acceleration or deceleration of HDX in each residue (schematically indicated with an *arrow*) was defined using the signal intensity ratio of each measurement to that of GC (*I*/*I*_(*GC*)_; see Materials and methods for details). When *I*/*I*_(*GC*)_ was decreased or increased (i.e., HDX was accelerated or decelerated) after subterahertz radiation or heating above the measurement error range, the corresponding residue was colored orange or blue, respectively. Dark and light colors indicate that the acceleration or deceleration of HDX was detected at 6 and 12 min and only at 12 min, respectively. Note that 3 min was selected as the reference time point at which the sample temperature reached plateau after subterahertz irradiation or heating. Error bars are derived from signal/noise ratio, following Eq. 6.

Next, the selected 21 residues classified as significantly accelerated or decelerated after subterahertz irradiation or heating (Fig. 2, *inset*) were mapped onto the Ub structure (Fig. 2; Fig. S6). We observed that irradiation at 18 mW/cm^2^ accelerated HDX in the residues located in the inner helical surface (Fig. 2, *left*; V26 and I30) and the hydrophobic surface composed of four *β*-strands, *β*1/*β*2 and *β*3/*β*5 (Fig. 2, *left*; I3, F4, L15, V17, I44, and V70), whereas it decelerated HDX in residues located in the surface loop and 3_10_ helix (Fig. 2, *left*; D21, Y59, and I61). Notably, these effects were qualitatively contradictory to those of temperature rise by 5°C; thus, the inner helical and hydrophobic patch regions were characterized by decelerated HDX, and the surface loop and 3_10_ helix regions were characterized by accelerated HDX (Fig. 2, *right*). Moreover, irradiation at 90 mW/cm^2^ with the same 5°C rise in temperature appeared to counteract the subterahertz-radiation-induced effects because the entire profile of HDX changes occurred approximately in the middle or mixture of these two effects (Fig. 2, *middle*). Therefore, it is unlikely that the temperature increase induced by subterahertz irradiation altered Ub dynamics. Alternatively, subterahertz energy could have directly excited the hydrogen-bond networks (i.e., intermolecular motions) of the protein and surrounding water molecules with various microscopic properties.

The subterahertz-radiation- and heat-induced effects observed in the HDX profiles may be overestimated because of the variation in time intervals between the subterahertz (or heat) perturbation and NMR probing of each sample (Fig. S2). This variation can affect the extent of HDX, depending on the rate. However, it was verified by the experimentally determined HDX rates for the GC sample that the history of the subterahertz-radiation- or heat-induced effect was reasonably maintained in their HDX profiles (Fig. S5). Moreover, in several residues with relatively fast HDX rates, the opposite effects observed between subterahertz irradiation and heating were underestimated by the variation in the perturbing and probing intervals (Fig. S7).

To determine whether the 6–10 h period of storage at 4°C (because of the long physical distance between the subterahertz-klystron and NMR devices; see Materials and methods) after irradiation could produce artifacts in the HDX data, we performed another THz-HDX experiment with an IMPATT-diode-based 0.1-THz light source installed near the NMR spectrometer. This experimental setup allowed us to substantially shorten the time interval to 12 min at room temperature (Fig. S8 *A*). The results showed that this subterahertz irradiation for 3 min without long-term storage caused an effect similar to that of klystron-based irradiation, i.e., an effect opposite to that of the temperature rise in the HDX profile (Fig. S9). Details of the results and experimental setup are shown in the Supporting materials and methods and Fig. S8.

## Discussion

We interpreted the influence of subterahertz irradiation on HDX kinetics in Ub as a type of solvent effect, which depends on its structure and the surrounding hydrogen-bond networks. This interpretation is supported by the strong pH dependence of the HDX rate and lack of partial denaturation of Ub during lyophilization (Supporting materials and methods; Figs. S3 and S10). Their experimental verifications are essential to explain the phenomena in line with the framework of a well-established kinetic model of HDX; protonated backbone amides (NH) involved in hydrogen-bonded secondary or tertiary structures or sequestered within the protein can be exchanged with the solvent deuterons in an open state to the solvent (37,56), as indicated by the kinetic scheme described by Englander’s group (57).

(7) $$NH\left(closed\right)\;\overset{K_{op}}{⇌}NH\left(open\right)\overset{k_{ch}}{\to }exchanged.$$

In typical HDX experiments for native proteins, the rate-limiting step is mostly observed during chemical exchange, and structural opening and closing are regarded as pre-equilibrium. This is known as the EX2 limit, and it is distinct from the alternative EX1 limit at which the HDX rate is determined by the opening rate (58). In the EX2 limit, the exchange rate constant of any hydrogen (*k*_*ex*_) is determined by its chemical exchange rate constant in the open form (*k*_*ch*_) multiplied by the equilibrium opening constant (*K*_*op*_), as follows (57):

(8) $$k_{ex}=K_{op}k_{ch}.$$

In our measurement, the HDX rates of the selected 21 Ub residues were strongly affected after a pH change from 5.6 to 7.2 (Fig. S10), consistent with the view that the HDX rates of these residues generally follow EX2 behavior (59). However, the slow HDX residues primarily located in the protein interior and the hydrophobic surface (e.g., Q41, R42, and L43 located in *β*3) were much less sensitive to the pH change than a 10-fold increase per pH unit expected for an ideal EX2 exchange rate (58). Such structural regions are rigid and less frequently open owing to intramolecular hydrogen-bond networks that are found between two *β* strands and hydrophobic interactions (30). This suggests that in these residues, protein folding (opening constant, *K*_*op*_) significantly affected the HDX rates (*k*_*ex*_).

Our NMR measurements showed that in the protein interior and hydrophobic surface (hydrophobic regions), HDX decelerated as the temperature increased from 25 to 30°C (Fig. 2, *right*). This indicates that the temperature rise by 5°C could optimize the conformation that makes the hydrogen-bond network surrounding the structural regions more rigid (smaller *K*_*op*_), thereby reducing the accessibility of the local solvent, D_2_O, to the open form of the protein. In fact, some residues in the *β*-sheet of Ub, including I44 and V70, may be associated with cold denaturation, whose backbone hydrogen bonds become longer under supercooled conditions (60). Conversely, HDX of the surface loop and short 3_10_ helix regions (hydrophilic regions) became rapid with the temperature rise. This is likely because the chemical exchange rate is little affected by the protein folding (*k*_*ex*_ ≈ *k*_*ch*_) and thus is increased by the temperature rise as expected only from the activation energy determining *k*_*ch*_ (59).

The opposite effect of the temperature rise on HDX kinetics was observed with 0.1-THz irradiation. This can be explained by a solvent effect that can lead to the following: 1) a larger *K*_*op*_ for the hydrophobic regions and 2) a smaller *k*_*ch*_ for the hydrophilic regions where *k*_*ex*_ ≈ *k*_*ch*_ is expected. The hydrophobic regions will be structurally unstable (larger *K*_*op*_) when subterahertz radiation induces the recombination of intramolecular hydrogen bonds with the intermolecular bonds in the presence of the solvent D_2_O. In contrast, the hydrophilic regions would be dominated by intermolecular hydrogen bonds with the solvent even before subterahertz irradiation. In fact, bound or slow water has been detected in the regions by solution NMR studies using reverse micelle encapsulation (39) and by molecular dynamics simulations (40). Thus, *k*_*ch*_ would be smaller if the subterahertz excitation blocked the access of the solvent D_2_O to the protonated backbone amides for the exchange via inducing a more extended hydrogen-bond network surrounding the hydrophilic regions. Although we could not determine the underlying mechanism, the number of recombination trials of the hydrogen-bond network required for its extension will be significantly increased on the 10-ps timescale by the subterahertz excitation. In this scenario, it may not be necessary to assume an extremely long residence time of a particular bound water, which contradicts previous views on Ub dynamics (61).

## Conclusion

To date, limited information is available regarding whether an externally applied alternating electromagnetic field with subterahertz frequency can directly and nonthermally influence protein and hydration dynamics, thus affecting biological functions. In this study, we investigated the difference between subterahertz-radiation- and heat-induced effects on the dynamics of Ub by developing a THz-HDX approach that combines klystron-based 0.1-THz irradiation with NMR-based HDX measurement. Using this method, we demonstrated that 0.1-THz irradiation affected Ub dynamics in a manner reflecting the heterogeneous nature of the hydrogen-bond network around the protein. Interestingly, the effect observed after 0.1-THz irradiation was opposite to that observed after temperature increase, suggesting that the applied 0.1-THz radiation energy was retained in specific protein and water interactions. Our results will help to expand our understanding of the relationship between the microscopic properties of fast-fluctuating dynamics and slow biological functions. Future studies should focus on elucidating the mechanism through which 0.1-THz excitation at the 10-ps timescale can influence the protein and hydration interactions of much slower time domains and cause an effect opposite to heating.

## Author contributions

M.I., Y. Tokunaga, K.T., and M.T. designed the research. Y. Tokunaga, M.I., M.T., H.I., M.K., Y. Tojima, and K.T. performed the research. Y. Tokunaga analyzed the data. M.I. and Y. Tokunaga interpreted the data and wrote the manuscript, which was edited by all authors.

## Supporting material

Document S1. Supporting materials and methods and Figs. S1–S10

Document S2. Article plus supporting material
