# Supplementary material for: Nonthermal excitation effects mediated by sub-terahertz radiation on hydrogen exchange in ubiquitin
Source: Biophys J. 2021 May 1;120(12):2386–93. doi: 10.1016/j.bpj.2021.04.013 (PMC8390810; doi:10.1016/j.bpj.2021.04.013)
Supplement: Document S1. Supporting materials and methods and Figs. S1–S10 [file mmc1.pdf]

Biophysical Journal, Volume *120*

Supplemental information

Nonthermal excitation effects mediated by sub-terahertz radiation on hydrogen exchange in ubiquitin

Yuji Tokunaga, Masahito Tanaka, Hitoshi Iida, Moto Kinoshita, Yuya Tojima, Koh Takeuchi, and Masahiko Imashimizu

Supplementary Text

The effect of lyophilization on the native Ub structure.

In addition to lyophilization-dissolution (LD), real-time HDX experiments were also initiated by buffer exchange via dilution, followed by concentration with ultrafiltration (UF). Although lyophilization could induce protein unfolding or structural collapse (54), we selected the LD procedure because of the following reasons: (i) LD requires a shorter time before NMR measurement and thus enhances reproducibility, and (ii) Ub is a highly stable protein. We compared the HDX profiles of Ub initiated by LD and UF procedures, which showed overlapping chemical shift patterns in the two spectra (Supplementary Fig. S3A). Moreover, the rate constants of the eight residues that could be determined under the experimental condition were nearly identical between the two experiments (Supplementary Fig. S3B). Therefore, we concluded that no denaturing effect occurred during lyophilization.

Method of THz-HDX experiment using an IMPATT diode-based light source

Procedures for preparation of lyophilized Ub, initiation of HDX by dissolving in D_2O, sub-THz irradiation, transfer to an NMR tube, and acquiring and processing NMR data are the same as those described for the THz-HDX experiments using the klystron-based light source, except followings: (i) Duration at room temperature was prolonged from 14 min to 62 min, in which sub-THz was irradiated for 3 min or 60 min. (ii) A continuous-wave IMPATT diode (Terasense, San Jose, CA) was used as a portable 0.1 THz light source (see the legend of Supplementary Fig. S8 for the details). This source can generate 96.9 GHz radiation and 181 mW power, which was pulse-modulated at a 50-Hz repetition rate, and a 20-ms pulse width of a square wave. The power density of the 0.1-THz radiation transmitted to the sample surface was measured as described in Materials and Methods, which was estimated to be 24 mW/cm^2. (iii) Delay of NMR measurement after sub-THz irradiation was shortened from 6-10 h to 12 min. This was made possible by using the portable IMPATT diode-based light source in AIST Tokyo Waterfront, where the NMR spectrometer is placed.

Result of THz-HDX experiment using an IMPATT diode-based light source

We investigated whether the 6-10 h delay of NMR measurement after sub-THz irradiation can significantly affect our interpretation of the sub-THz-induced effect on HDX kinetics. In order to shorten the delay, we constructed another experimental setup for THz-HDX system with a portable 0.1 THz light source (IMPATT diode device) that was installed near the NMR spectrometer. Using the IMPATT diode device, we applied 0.1-THz radiation with a power density of 24 mW/cm^2 to the Ub solution for 3 min or 60 min, in the same manner as the klystron-based experiment (Supplementary Fig. S8A), except that (i) the delay of NMR

measurement was 12 min and (ii) buffer pH was decreased from 6.4 to 5.6. The acidic pH generally decreases the chemical exchange rate (57-59), allowing a prolonged sub-THz irradiation during HDX at room temperature. HDX reactions were then measured by NMR after a total of 74 min of Ub dissolution in D_2O (Supplementary Fig. S8A).

As similarly observed in klystron-based irradiation at 18 mW/cm^2, the IMPATT-diode-based irradiation at 24 mW/cm^2 for 3 min induced changes in HDX kinetics opposite to those induced by increased temperature (compare Supplementary Fig. S8 B and C with Fig. 2). Notably, the irradiated HDX profiles negatively correlates with the non-irradiated profile with 5 °C temperature rise (TC), irrespective of the types of the sub-THz-sources and the delay of NMR measurement (Supplementary Fig. S9). In contrast, when the irradiation duration was increased to 60 min, the HDX profile of IMPATT-diode-based irradiation was similar to (i.e., positively correlates with) that of TC (Supplementary Fig. S9). The IMPATT-diode-based irradiation for 3 min and 60 min increased volume-averaged sample temperatures by 0.5 °C and 2 °C, respectively, with a plateau of approximately 20 min. The relatively enhanced temperature rise per power density compared to the klystron-based irradiation is due to its much longer pulse duration (i.e. less likely thermal diffusion per duty cycle). Therefore, the sub-THz-induced nonthermal effect could be dominated by the effect of heat dissipated via the prolonged irradiation for 60 min. This result is also consistent with the fact that the high-power klystron-based irradiation at 90 mW/cm^2, which increased the sample temperature by 5 °C, made the nonthermal effect on the HDX profile closer to the heating effect (Fig. 2).

Supplementary Figures

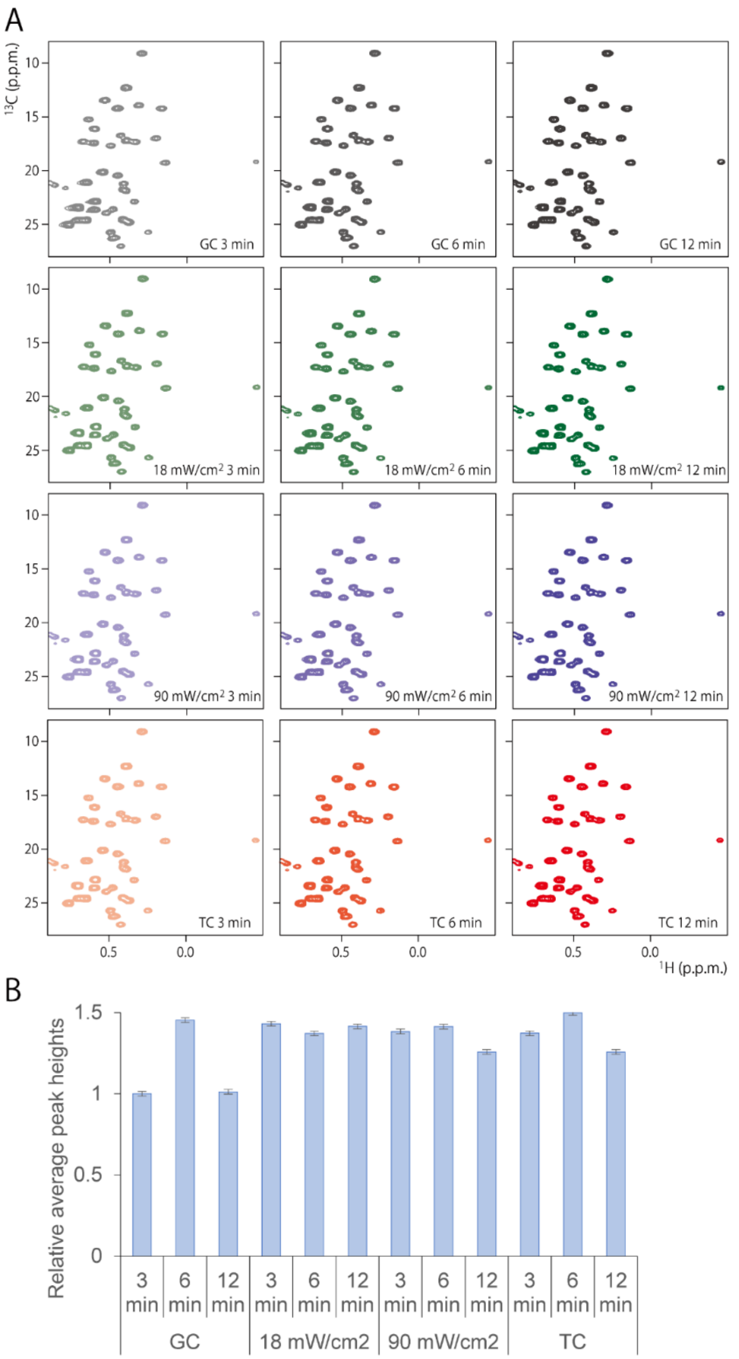

Supplementary Fig. S1. Normalization of amide signal peak heights using those from unexchangeable proton sites. (**A**) ^{1}H-^{13}C constant-time HSQC spectra of THz-HDX samples. Peak heights of 37 methyl resonances were analyzed. (**B**) Relative average peak height of 37 methyl resonances of each sample, in which GC-3 min is set to 1. Error bars are derived from signal-to-noise ratios. Assuming that these values reflect the differences in Ub concentration among samples, peak heights of amide signals were divided by these values to exclude the effect of such differences.

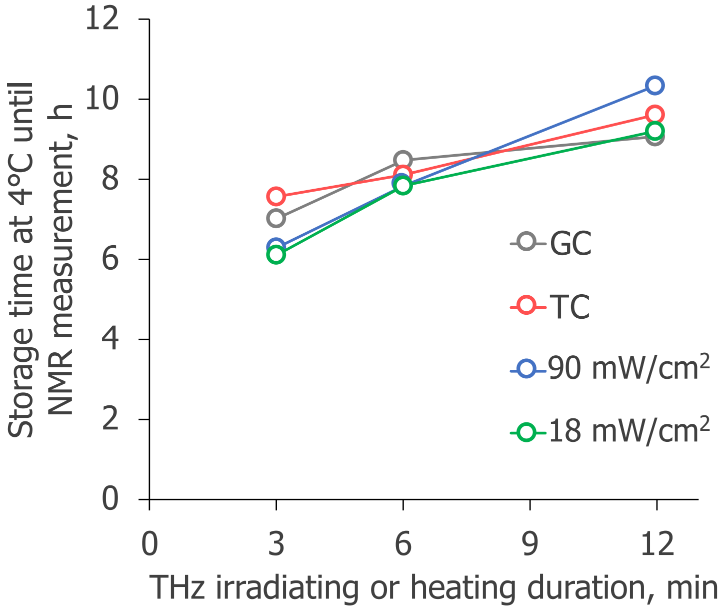

Supplementary Fig. S2. Variation in storage times at 4 °C until NMR measurement (i.e., variation in time intervals between the THz/heat perturbations and NMR probing) of each sample. GC and TC represent the general control experiment and the temperature control experiment, respectively.

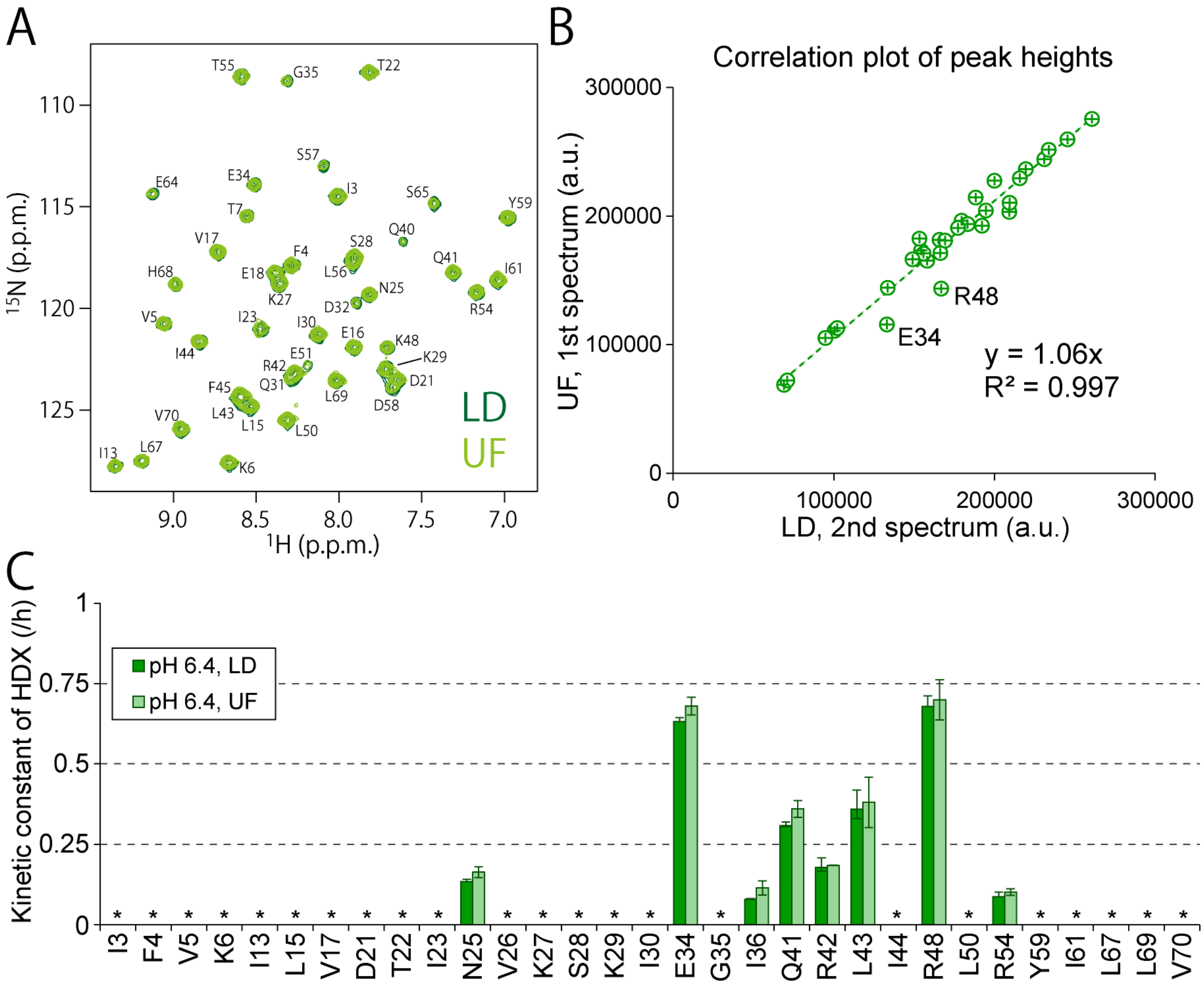

Supplementary Fig. S3. HDX profiles of Ub prepared by lyophilization-dissolution (LD) and buffer exchange by ultrafiltration (UF). (**A**) Overlaid amide ^{1}H-^{15}N SOFAST-HMQC spectra of Ub prepared by LD (deep green) and UF (light green), both of which were measured at 48 min (LD) and 51 min (UF) after exchanged into D_2O. NMR experiments were performed at 277 K in a 700 MHz magnet. (**B**) Correlation plot of peak heights between spectra shown in (**A**). Two remarkable outliers from the linear fit, E34 and R48, would reflect in the slight difference of delays before measurement (longer in the UF experiment by 3 min), as these two residues have the largest exchange rates as shown in (**C**). Error bars are based on noise levels. (**C**) HDX rate constants determined from LD and UF data. The mean values of duplicate experiments ± the differences are shown. HDX of the residues labeled with asterisks was too slow to determine the rate constant from the experimental condition.

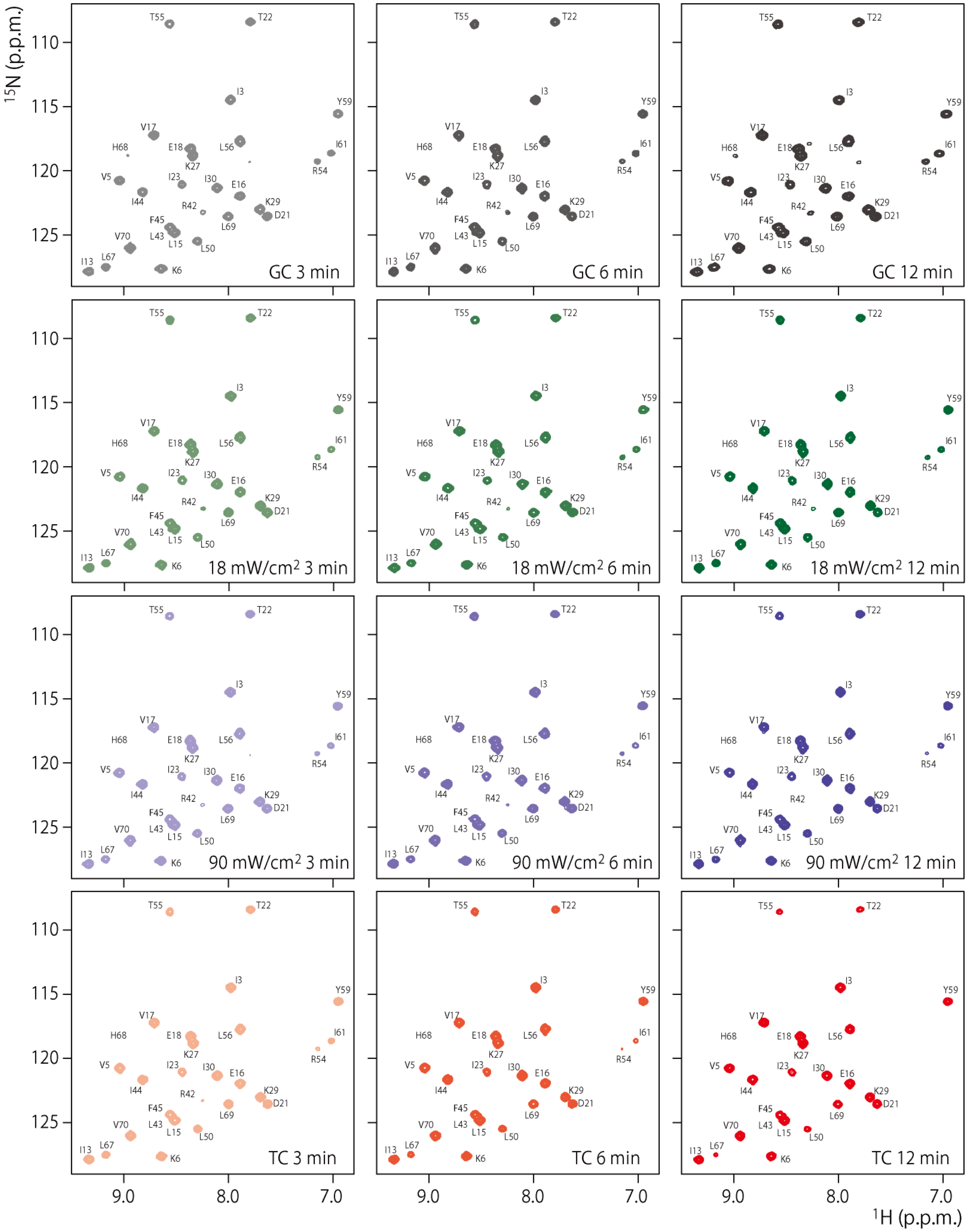

Supplementary Fig. S4. Amide ^{1}H-^{15}N SOFAST-HMQC spectra of THz-HDX samples.

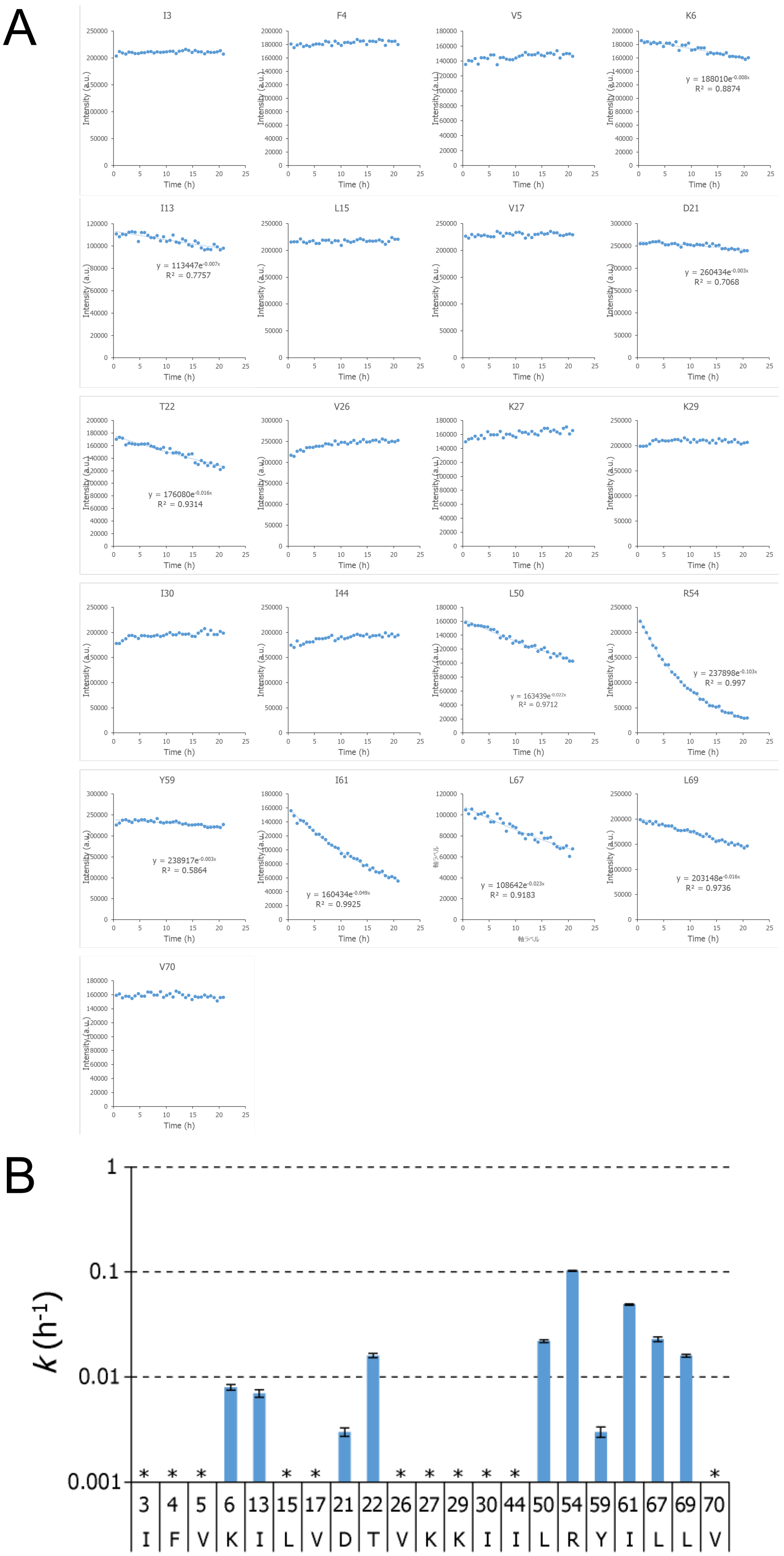
A
I3
F4
V5
K6
y = 188010e-0.008x
R² = 0.8874
I13
y = 113447e-0.007x
R² = 0.7757
L15
V17
D21
y = 260434e-0.003x
R² = 0.7068
T22
y = 176080e-0.016x
R² = 0.9314
V26
K27
K29
I30
I44
L50
y = 163439e-0.022x
R² = 0.9712
R54
y = 237898e-0.103x
R² = 0.997
Y59
y = 238917e-0.003x
R² = 0.5864
I61
y = 160434e-0.049x
R² = 0.9925
L67
y = 108642e-0.023x
R² = 0.9183
L69
y = 203148e-0.018x
R² = 0.9736
V70
Intensity (a.u.)
Time (h)
B
k (h-1)
1
0.1
0.01
0.001
3 4 5 6 13 15 17 21 22 26 27 29 30 44 50 54 59 61 67 69 70
I F V K I L V D T V K K I I L R Y I L L V

Supplementary Fig. S5. (**A**) Real-time NMR measurement of HDX in a GC sample. HDX profiles of the selected 21 amino acid residues were measured by NMR experiments just after dissolution of lyophilized Ub in D_2O. We used the same sample conditions, preparation procedure, and NMR experimental conditions as those used in the THz-HDX experiment, except that the NMR experiment was initiated 13 min after the dissolution and that spectra were acquired by Bruker Avance 700 Spectrometer equipped with a TXI triple resonance probe in 35 min 51 s per spectrum with 24 scans. V5, V26, K27, K29, I30, and I44 showed more than 3% increase in signal intensity. This might be due to decrease in transverse relaxation rates upon HDX of nearby labile proton sites. (**B**) The residue-specific rate constant of HDX, k, was obtained by fitting the data to an exponential decay equation $I = I_0 exp(-kt)$, where I_0 stands for the initial signal intensity without HDX. The residues were classified into three groups with the different rate constant k: I3, F4, V5, L15, V17, V26, K27, K29, I30, I44, and V70 have $k \approx 0$; T22, L50, L67, and L69 have $k \sim 0.02\ h^{-1}$; R54 and I61 have $k \sim 0.1\ h^{-1}$. Thus, in the first group with the smallest k, only less than 3% of signal intensity was reduced over the measurement time of ca. 20 h. Owing to the relatively large k, the THz/heat-induced effects on HDX were underestimated in several residues of the remaining groups (see Supplementary Fig. S7). Error bars correspond to errors of curve fitting by the exponential decay. HDX of the residues labeled with asterisks was too slow to determine the exchange rate under the experimental condition.

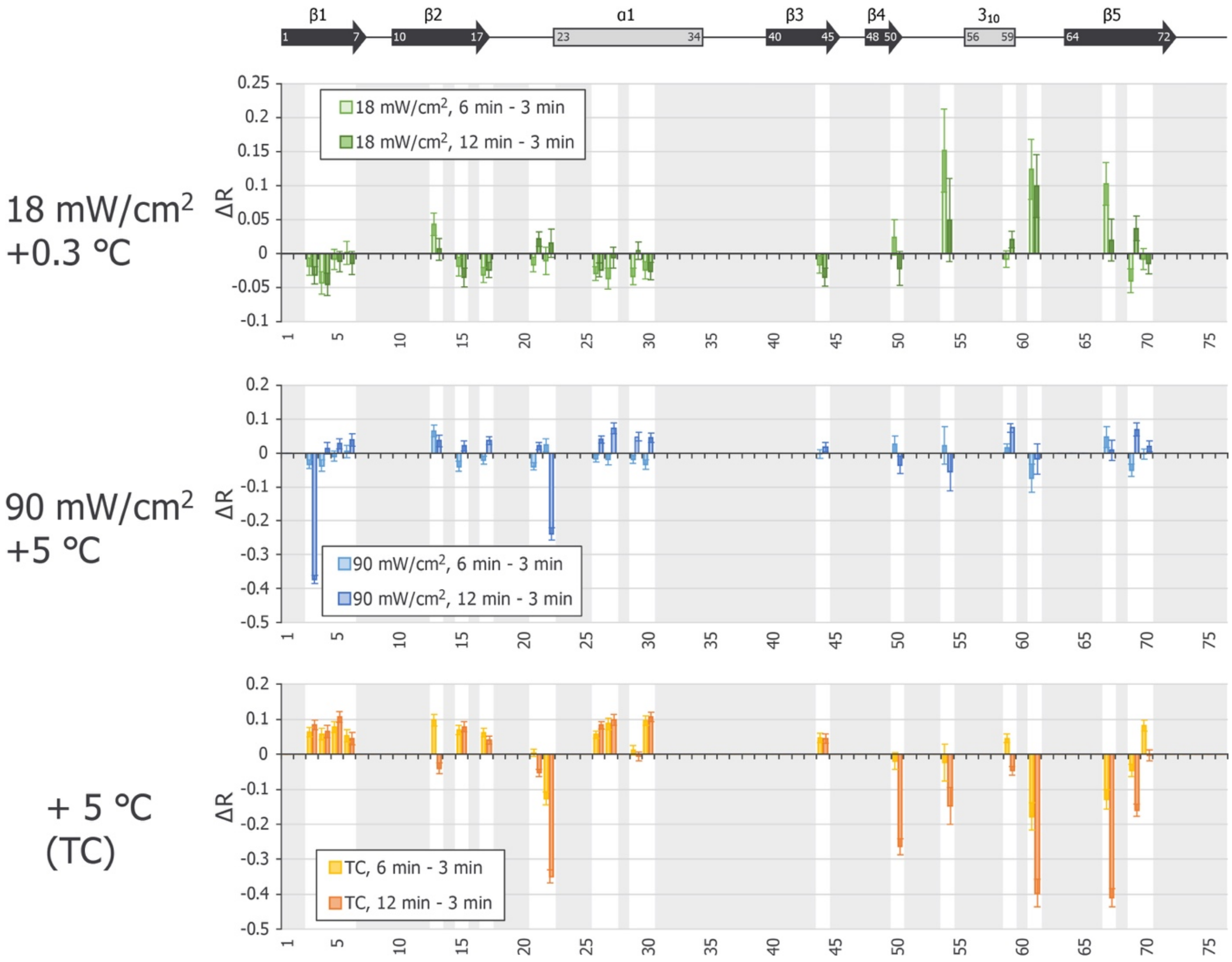

Supplementary Fig. S6. Effect of THz irradiation at low (top) or high (center) power density and the effect of temperature increase (bottom) on HDX changes through all amino acid residues of Ub. The secondary structure of Ub is also shown at the top of the graphs. TC represents the temperature control experiment. The magnitude of the effect (y-axis) is defined as ΔR, where R is the signal intensity ratio of each measurement to GC's measurement ($I/I_{(GC)}$). GC represents the general control experiment. ΔR is a difference in R at 6 or 12 min from that at 3 min. The residues excluded from the analysis (see Materials and Methods for the details) are indicated by gray color.

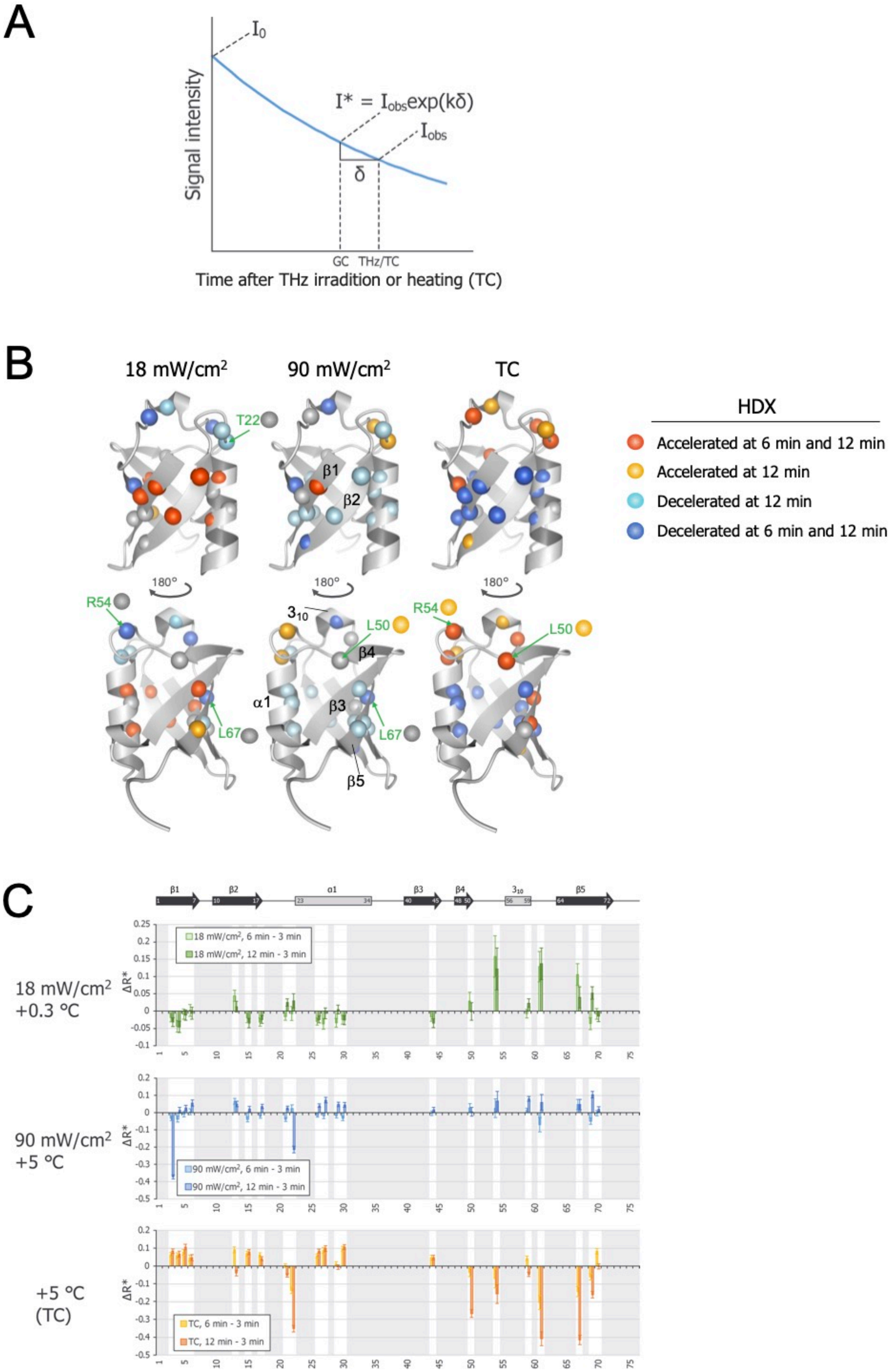
A
I_0
$I^* = I_{obs}\exp(k\delta)$
I_{obs}
δ
Signal intensity
GC
THz/TC
Time after THz irradition or heating (TC)
B
18 mW/cm²
90 mW/cm²
TC
T22
β1
β2
180°
R54
3₁₀
L50
β4
α1
β3
L67
β5
HDX
Accelerated at 6 min and 12 min
Accelerated at 12 min
Decelerated at 12 min
Decelerated at 6 min and 12 min
C
β1
β2
α1
β3
β4
3₁₀
β5
18 mW/cm², 6 min - 3 min
18 mW/cm², 12 min - 3 min
18 mW/cm²
+0.3 °C
ΔR*
90 mW/cm², 6 min - 3 min
90 mW/cm², 12 min - 3 min
90 mW/cm²
+5 °C
TC, 6 min - 3 min
TC, 12 min - 3 min
+5 °C
(TC)

Supplementary Fig. S7. Effect of sub-THz irradiation at low (left) and high (middle) power density and temperature increase (right, TC) on the amid proton exchange of Ub, in which the difference in the time intervals between the sample correction and NMR measurement (i.e. the interval between sub-THz/heat perturbations and NMR probing) were corrected. (**A**) The signal intensities of the sub-THz-irradiated and TC samples were corrected to have the perturbing and probing intervals equal to those of the GC samples. The corrected intensity I^* was estimated by an equation $I^* = I_{obs}exp(k\delta)$, where I_{obs} is the observed intensity, δ is the interval difference (Supplementary Fig. S2), and k is the residue-specific rate constant of HDX (Supplementary Fig. S5B). The corrected ratio difference ΔR^* was determined using I^*, according to equations (1) and (2) (see Materials and Methods). The ΔR^* profiles in the selected 21 residues were then mapped onto the tertiary (**B**) and secondary (**C**) structures of Ub, in the same manners as shown Fig. 2 and Supplementary Fig. S6, respectively. In the panel B, the difference between ΔR and ΔR^* profiles are indicated with arrows, the residue numbers, and the labels showing acceleration, deceleration, and no significant change in Fig. 2. As shown in the panel B, the correction of the signal intensity to produce the ΔR^* profiles successfully uncovered the sub-THz wave- or heat-induced changes in several additional residues (T22, R54, and L67 in 18 mW/cm^2, L50 and L67 in 90 mW/cm^2, and L50 and R54 in TC) that were not able to be detected in the uncorrected ΔR profiles, therefore allowed us to more prominently capture the opposite effect observed between the sub-THz irradiation and heating.

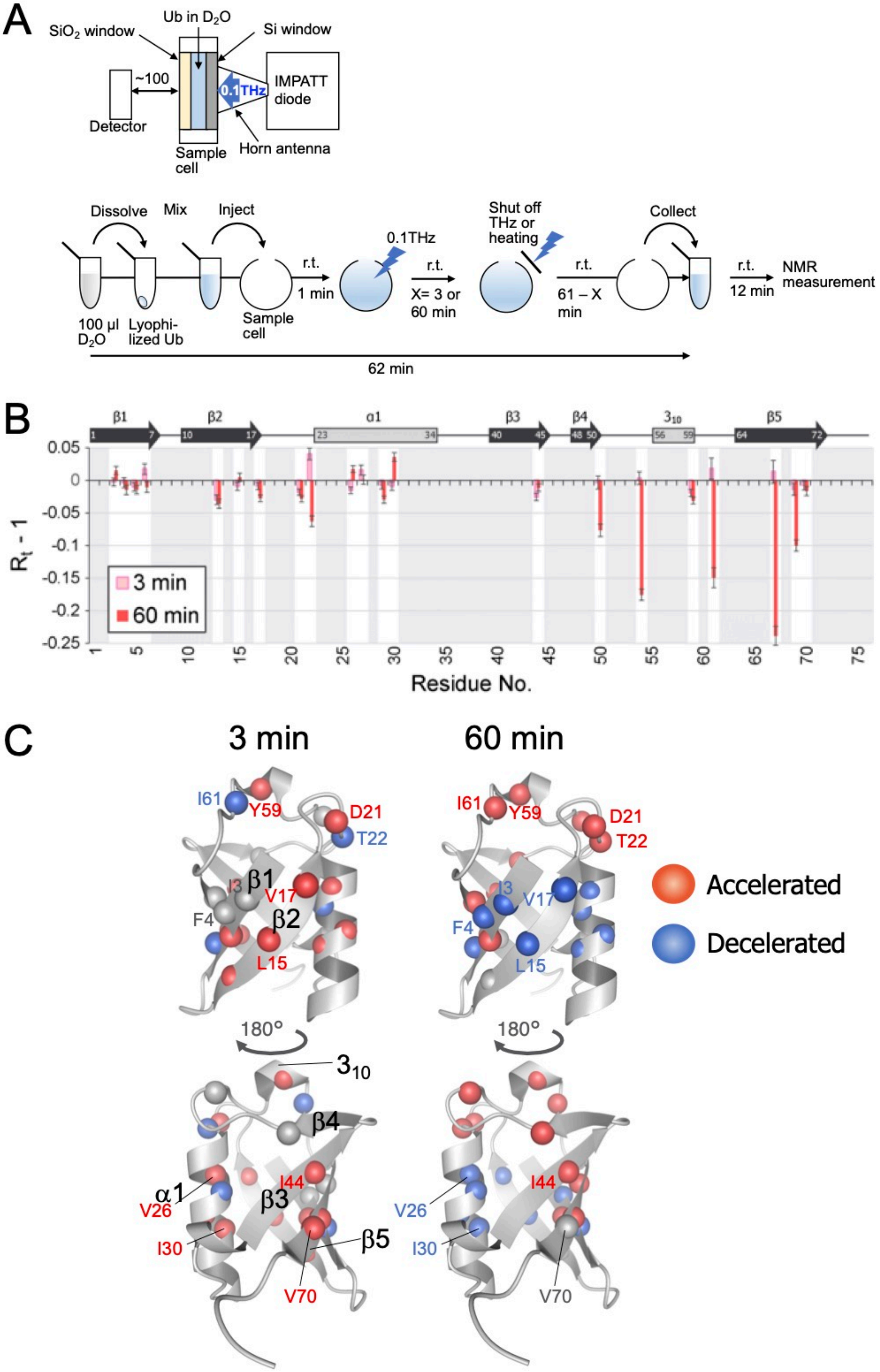

A
SiO2 window
Ub in D2O
Si window
~100
0.1THz
IMPATT diode
Detector
Sample cell
Horn antenna
Dissolve
Mix
Inject
0.1THz
Shut off THz or heating
Collect
r.t.
1 min
X= 3 or 60 min
61 – X min
12 min
NMR measurement
100 µl D2O
Lyophi-lized Ub
Sample cell
62 min
B
Rt - 1
3 min
60 min
Residue No.
C
3 min
60 min
180°
Accelerated
Decelerated

Supplementary Fig. S8. THz-HDX without a long interval between sub-THz perturbation and NMR probing**.** (**A**) Sub-THz irradiation to Ub solution using IMPATT diode-based light source. Details of the light source are described in Supplementary Methods. The length unit is shown in millimeter. Sample cell and detector are the same as those shown in Fig. 1A. The experimental procedure of the THz-HDX using the IMPATT diode device is schematically shown on the bottom (see the legend of Fig. 1C for more details). (**B**) Effect of sub-THz irradiation for 3 min (pink) or 60 min (red) on HDX through all amino acid residues of Ub. The secondary structure of Ub is also shown at the top of the graphs. The residues excluded from the analysis are indicated by gray color. The magnitude of the effect (y-axis) is defined as $R_t - 1$, where R_t is the signal intensity ratio of each measurement to GC's measurement ($I_{(THz)}/I_{(GC)}$), as defined by Eq. (1). GC represents the general control experiment. For the analysis of HDX data, we used $R_t - 1$, instead of ΔR (see Eq. (2)) used for analyzing the klystron data in Fig. 2 and Supplementary Figs. S5 and S7. This is because temperature elevation in 3 min was as small as 0.5 °C in the IMPATT diode-based experiment, and thus $R_{3\,min}$ data per se would mainly reflect the nonthermal effect as observed in the klystron-based irradiation at 18 mW/cm^2. In contrast, the irradiation for 60 min resulted in accumulative temperature elevation of 2°C, which supposedly rendered the 60 min data dominated by the thermal effect, as indicated in Supplementary Fig. S9. (**C**) The tertiary Ub structures with 180° rotation are shown (PDB accession code: 1UBQ). Amide nitrogen atoms of the analyzed residues are shown. Amino acid residues were mapped in the Ub structure when the HDX of the main chain amide groups was accelerated or decelerated. The acceleration or deceleration of HDX in each residue was defined using the signal intensity ratio of each measurement to GC's measurement ($I/I_{(GC)}$; see Materials and Methods for details). When HDX was accelerated (i.e., $0 > -Error(R_t) > R_t - 1$) or decelerated (i.e., $R_t - 1 > Error(R_t) > 0$), following sub-THz radiation above the measurement error range, the corresponding residue was colored red or blue, respectively. $Error(R_t)$ values are derived from signal-to-noise ratio, following Eq. (6).

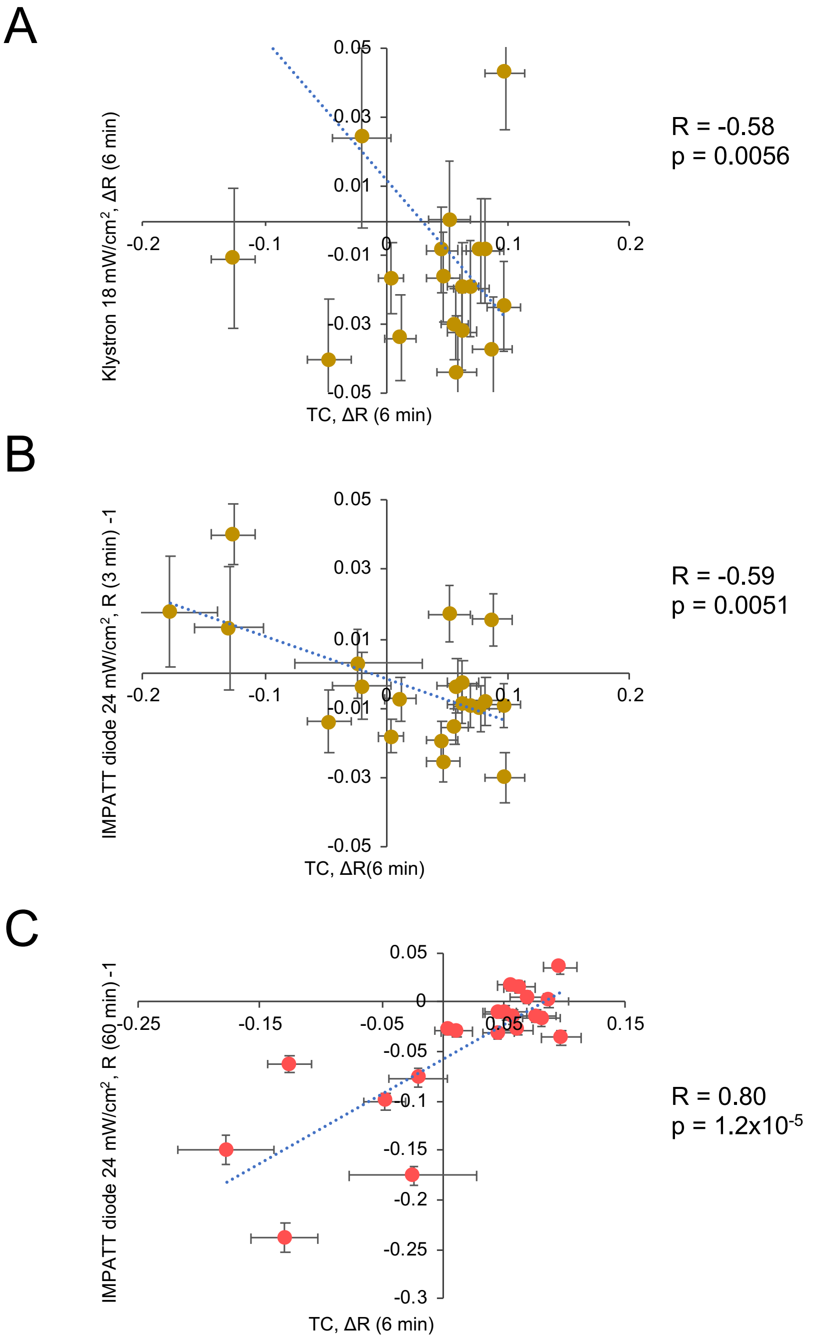

Supplementary Fig. S9. Correlation relationships of ΔR (or R_t -1, see Materials and Methods) between (**A**) klystron-based sub-THz irradiation at 18 mW/cm^2 for 6 min and TC for 6 min, (**B**) IMPATT diode-based sub-THz irradiation at 24 mW/cm^2 for 3 min and TC for 6 min, and (**C**) IMPATT diode-based sub-THz irradiation at 24 mW/cm^2 for 60 min and TC for 6 min. Error bars represent errors calculated with Eq. 5 (for the experiments with klystron device and TC) and with Eq. 6 (for the experiment with IMPATT diode device). Pearson correlation coefficient R between the two variables and the p-value are shown in each graph.

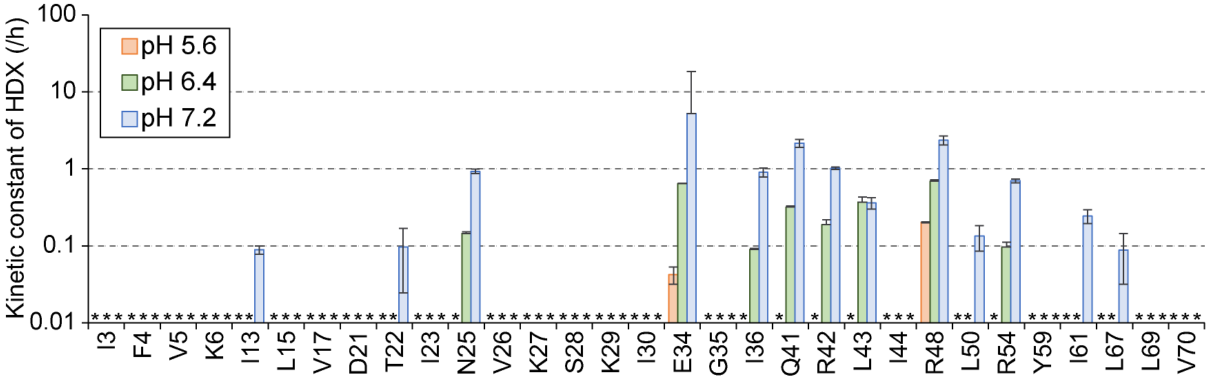

Supplementary Fig. S10. Rate constants for HDX under different pH conditions. The mean values of duplicate experiments ± the differences are shown. Experiments were performed at 277 K in a 700 MHz magnet. HDX of the residues labeled with asterisks was too slow to determine the rate constant under the experimental condition. Among 13 residues, whose rate constants were determined at pH 7.2, rate constants of 5 and 11 residues could not be determined at pH 6.4 and 5.6, respectively, due to the decelerated HDX.
