# Supplementary material for: Nonthermal excitation effects mediated by sub-terahertz radiation on hydrogen exchange in ubiquitin
Source: Biophys J. 2021 May 1;120(12):2386–93. doi: 10.1016/j.bpj.2021.04.013 (PMC8390810; doi:10.1016/j.bpj.2021.04.013)
Supplement: Document S2. Article plus supporting material [file mmc2.pdf]

Biophysical Journal
Biophysical Letter

Biophysical Society

Nonthermal excitation effects mediated by sub-terahertz radiation on hydrogen exchange in ubiquitin

Yuji Tokunaga,[1] Masahito Tanaka,[2] Hitoshi Iida,[3] Moto Kinoshita,[3] Yuya Tojima,[3] Koh Takeuchi,[1] and Masahiko Imashimizu[1,*]

[1]Cellular and Molecular Biotechnology Research Institute, National Institute of Advanced Industrial Science and Technology, Tokyo, Japan; [2]Research Institute for Measurement and Analytical Instrumentation and [3]Research Institute for Physical Measurement, National Institute of Advanced Industrial Science and Technology, Tsukuba, Japan

ABSTRACT Water dynamics in the hydration layers of biomolecules play crucial roles in a wide range of biological functions. A hydrated protein contains multiple components of diffusional and vibrational dynamics of water and protein, which may be coupled at ~0.1-THz frequency (10-ps timescale) at room temperature. However, the microscopic description of biomolecular functions based on various modes of protein-water-coupled motions remains elusive. A novel approach for perturbing the hydration dynamics in the subterahertz frequency range and probing them at the atomic level is therefore warranted. In this study, we investigated the effect of klystron-based, intense 0.1-THz excitation on the slow dynamics of ubiquitin using NMR-based measurements of hydrogen-deuterium exchange. We demonstrated that the subterahertz irradiation accelerated the hydrogen-deuterium exchange of the amides located in the interior of the protein and hydrophobic surfaces while decelerating this exchange in the amides located in the surface loop and short 3_{10} helix regions. This subterahertz-radiation-induced effect was qualitatively contradictory to the increased-temperature-induced effect. Our results suggest that the heterogeneous water dynamics occurring at the protein-water interface include components that are nonthermally excited by the subterahertz radiation. Such subterahertz-excited components may be linked to the slow function-related dynamics of the protein.

SIGNIFICANCE It has been shown that at physiological temperatures in aqueous solution, fluctuating dynamics of protein and coupled water molecules occur in the subterahertz frequency range. If so, does the externally applied alternating electromagnetic field with subterahertz frequency resonantly interact with the protein-water-coupled dynamics and nonthermally influence protein functions? Can we use subterahertz radiation energy as an efficient tool for understanding the microscopic details of elementary processes for biomolecular functions, including the contribution of water? We challenge this open question, combining the 0.1-THz irradiation with NMR-based measurements at the atomic level. In particular, we demonstrate that the applied subterahertz radiation energy leads to a solvent effect on structural dynamic changes of ubiquitin in a manner that cannot be explained by temperature increase.

INTRODUCTION

The hydration layers surrounding proteins play crucial roles in biological functions such as folding, enzymatic reactions, and protein-protein interactions (1–3). The basic mechanisms underlying water dynamics in the hydration layers of biomolecules have been extensively investigated using spectroscopic approaches and molecular dynamics simulations (4,5). These studies have revealed that diffusive protein and hydration dynamics occur on the same timescale on the order of 10 ps at physiological temperatures (6,7). Because protein and hydration dynamics show similar temperature dependency, a mechanism of coupled protein-hydration water relaxation has been suggested (6,7). On the same or slightly faster timescale, the low-frequency vibrational mode of proteins also overlaps with the diffusive protein and hydration dynamics (8,9). Such a variety of diffusional and vibrational modes of protein and water dynamics at this timescale would be directly linked to elementary molecular processes involved in the expression of biological functions. However, these dynamics show similar dielectric responses, making it difficult to derive a simplified description of biomolecular functions based on microscopic hydration properties (10,11). Indeed, such a direct link

Submitted December 1, 2020, and accepted for publication April 16, 2021.
*Correspondence: m.imashimizu@aist.go.jp
Editor: Wendy Shaw.
https://doi.org/10.1016/j.bpj.2021.04.013
© 2021 Biophysical Society.
This is an open access article under the CC BY license (http://creativecommons.org/licenses/by/4.0/).

between the fast dynamics and biological functions is not supported by a recent study of NMR relaxation (12).

The dynamics with a 10-ps timescale correspond to a frequency of 0.1 THz in an oscillation period. Oscillating protein motions of 0.1-THz resonant frequency were first described by normal mode analysis over 30 years ago (13). In an actual aqueous protein solution, water's rotational relaxations, rather than protein motions, are detected as the dominant dielectric responses at ~0.1 THz (8,14–17). However, as mentioned above, there are multiple superimposed components associated with protein and water motions in this frequency region at physiological temperatures. This prompted us to hypothesize that the 0.1-THz radiation may directly interact with biologically relevant protein-water-coupled motions observed at the same subterahertz frequency. More precisely, the subterahertz excitation energy is possibly redistributed to the hydrogen-bond network specific to the protein-water interface before thermal dissipation to the bulk water, thereby altering hydration dynamics. Notably, protein and hydration dynamics influence each other (8,10,11) and are strongly dependent on temperature (8,11). Therefore, subterahertz and thermal excitations might have nearly the same consequence unless the subterahertz excitation energy remains localized in the hydration dynamics largely beyond 10 ps. In other words, the subterahertz excitation energy must be adiabatically retained in the specific hydrogen-bond network localized and restrained at the protein-water interface until the timescales relevant to the structural changes of proteins.

Innovative approaches for analyzing biomacromolecular dynamics such as the optical Kerr effect, extraordinary acoustic Raman, and kinetic and anisotropic terahertz spectroscopies have been developed (9,18–21). Notably, these approaches revealed that collective water and biomolecule (protein or DNA) dynamics could persist well beyond the 10-ps timescale at physiological temperature, contributing to enzymatic activities on biological timescales (22–24). In addition, several studies using different (sub)terahertz light sources have shown that (sub)terahertz excitation alters the kinetics of DNA conformations and assemblies that are slow enough to be detected by conventional biochemical techniques (25). Moreover, it changes the electron density in the hydrated lysozyme crystal (26) and significantly affects actin polymerization (27) and gene expression (28) under physiological conditions. The above studies have performed well-designed control experiments that excluded the possible effect of (sub)terahertz-radiation-induced temperature increase. Thus, we can assume that the effect of subterahertz excitation on protein and hydration dynamics can be discriminated from the heating effect, and the difference would be reflected in the slow relaxation processes detectable by proton exchange reactions occurring on timescales as slow as minutes to hours at room temperature (29,30). The consequence of subterahertz excitation in the slow reactions occurring in an aqueous solution has not yet been directly assessed at the atomic level.

To monitor such slow relaxation processes after subterahertz or thermal excitation, we developed a novel, to our knowledge, method that combines a klystron-based 0.1-THz irradiation system (subterahertz-klystron) with NMR-based hydrogen-deuterium exchange (HDX), which we have termed THz-HDX. NMR-based HDX is a well-established technique for analyzing protein dynamics in aqueous systems (31,32). Herein, we used ubiquitin (Ub) as a model protein. Ub is a small, 76-amino acid globular protein found ubiquitously in all eukaryotic organisms (30,33), and it is extremely stable under heat denaturation and exhibits slow amide proton exchange kinetics (34,35). In addition, Ub has a tightly folded β-sheet, an α-helix, a short 3_{10}-helix, and surface loops between these elements in its compact structure, allowing physicochemical study of protein structure with high generality. Therefore, Ub has been intensively investigated for assessing conformational and hydrational changes upon external perturbations using NMR-based HDX and relaxation techniques (29,30,36–39), X-ray crystallography coupled with molecular dynamics simulations (40–42), and terahertz spectroscopy (43,44).

Using the THz-HDX method, we characterized the amide proton exchange kinetics of Ub after subterahertz irradiation and compared them with those after temperature increase mediated by heat conduction. We demonstrated that these two perturbations differently influenced the HDX in Ub. The subterahertz irradiation primarily increased the HDX of amides clustered in an interior region and a hydrophobic surface but decreased the amide HDX in the surface loop and short 3_{10} helix regions. We also found that such subterahertz-induced effects were qualitatively opposite to those induced by increased temperature. This result is consistent with the view that heterogeneous water and protein dynamics at the interface are nonthermally excited by subterahertz irradiation.

MATERIALS AND METHODS

Subterahertz source

Subterahertz irradiation experiments were performed using a klystron-based subterahertz source (45). A schematic representation of the experimental setup is illustrated in Fig. 1 *A*. The terahertz source comprised a W-band oscillator, a preamplifier, an isolator, a direct reading attenuator (CAR-1050-01; WiseWave, Torrance, CA), a klystron (Extended Interaction Klystron VKB2461; CPI, Palo Alto, CA), and a pyramidal horn antenna. All devices were connected through rectangular waveguides, WR-10. This source can generate 95 $\pm$ 0.25 GHz of radiation, a 10-kHz repetition rate, and a 0.8-μs pulse width of a square wave. The emitted subterahertz radiation is roughly collimated by a quartz plano-convex lens and monochromated by a bandpass filter with a center frequency of 95 GHz (MMBPF40; Joint Technology Development Platform, Kyoto, Japan). The subterahertz radiation diameter was estimated to be ~20 mm at the sample position by measuring the full width at half maximum. The sample solution was set at a distance of 200 mm from the bandpass filter (Fig. 1 *A*).

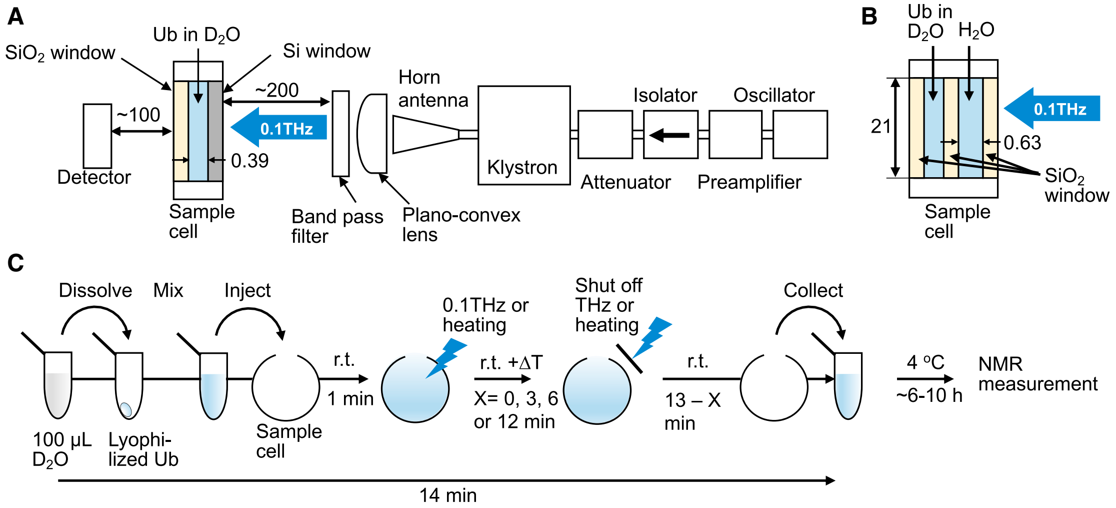

FIGURE 1 Schematic representation of subterahertz radiation experimental setup. (*A* and *B*) Subterahertz irradiation (*A*) and subterahertz-dependent heat conduction (*B*) to ubiquitin (Ub) solution using the same klystron-based subterahertz source. Devices for subterahertz radiation were connected through rectangular waveguides, WR-10. The length unit is shown in millimeter. See Materials and methods for details. (*C*) THz-HDX experiment. Lyophilized Ub was dissolved in 100 μL D_2O to obtain 0.5 mM Ub solution, which was thoroughly mixed using a vortex mixer and was then injected into the sample cell within 1 min at room temperature (rt) of ~25°C. The sample was incubated for 13 min at rt and was subjected to temperature increase (+ΔT) caused by subterahertz radiation or heat conduction for a variable time (X = 0, 3, 6, or 12 min). The experiment conducted at X = 0 corresponds to control. After 14 min of dissolution in D_2O, Ub solution was collected in the sample tube and stored at 4°C until NMR-based HDX measurement was performed. The time interval between the sample collection and NMR measurement per condition is shown in Fig. S2.

The maximal power density of the 0.1-THz radiation that was irradiated on the sample with an effective diameter of 21 mm was estimated to be 250 mW/cm^2 using a sensitive thermal sensor (3A-P-THz; Ophir Optronics Solutions, Jerusalem, Israel), which is specific for terahertz and subterahertz radiation. The efficiency of 0.1-THz radiation was determined by comparing the radiation powers measured using a reference calorimeter (46) and the detector. The attenuator controls the power density. The subterahertz power was monitored using the detector set downstream of the sample cell during the irradiation experiment.

Subterahertz irradiation and heat treatment of Ub

Lyophilized Ub was dissolved in 100 μL of D_2O, thoroughly mixed using a vortex mixer, and then injected into a demountable liquid transmission cell (DLC-M25; Harrick Scientific Products, Ossining, NY) within 1 min at room temperature (~25°C; Fig. 1 *C*). The pathlength of the D_2O sample containing Ub was adjusted to 390 μm using a Teflon spacer. A high-resistivity float zone silicon window (Tydex, St. Petersburg, Russia) and SiO_2 window (QPSQ-25C02-10-5; SIGMAKOKI, Tokyo, Japan), each with a thickness of 2.0 mm and an effective diameter of 21 mm, were placed upstream and downstream of the sample cell, respectively (Fig. 1, *A* and *B*). The conditions of the sample solution were visually identified through the SiO_2 window. Approximately 40% of the radiation was lost owing to reflection on the silicon window; therefore, the remaining 60% of the subterahertz radiation was transmitted to the aqueous sample. Consequently, the low- and high-power density of subterahertz radiation transmitted to the sample surface was estimated to be 18 and 90 mW/cm^2, respectively, each of which was used as the subterahertz exposure condition. Owing to the large absorption coefficient of water, 97% of the transmitted radiation was absorbed by the 390-μm-thick sample solution ($\alpha = 83\ cm^{-1}$ at 25°C, 0.1 THz) (45). The Ub sample was exposed to either subterahertz radiation or heating (terahertz radiation-derived heat conduction) using the same klystron-based 0.1-THz source under ambient conditions (~25°C) (Fig. 1, *A* and *B*). For heating, the subterahertz wave was irradiated onto 630-μm-thick water (~220 μL) inserted between two SiO_2 windows (Fig. 1 *B*). The temperature of the sample was measured using a K-type thermocouple.

Preparation of isotope-labeled yeast Ub

Uniformly $^{13}C/^{15}N$-labeled recombinant yeast Ub was prepared as previously described (47). Briefly, *Escherichia coli* BL21(DE3) cells were transformed with the plasmid vector pET-26b (Merck Millipore, Burlington, MA), harboring the gene encoding Ub (76 amino acids) at *Nde*I/*Sal*I restriction sites, without additional sequences. Five colonies of transformed cells cultured on a Luria-Bertani-agarose plate containing 50 μg/mL kanamycin were inoculated into 10 mL of Luria-Bertani-kanamycin medium in a 50-mL conical tube and then incubated by shaking at 37°C for 6 h. Next, the cell pellet obtained after centrifugation was resuspended in 500 mL of M9-kanamycin medium in a 2-L Sakaguchi flask, containing [^{15}N] ammonium chloride and [$^{13}C_6$] D-glucose as the sole nitrogen and carbon sources, respectively, and then incubated while shaking at 37°C. When the optical density at 600 nm reached 0.6–0.8, isopropyl-β-D-1-thiogalactopyranoside was supplemented at a concentration of 1 mM to induce the overexpression of Ub and further incubated for 6 h. Cells were harvested by centrifugation and stored at −80°C until purification. The cell pellet was resuspended in 30 mL lysis buffer containing 50 mM sodium acetate at pH 5.5. Cells were lysed by sonication on ice, and the cell debris was removed by centrifugation at 20,000 × *g* at 8°C for 1 h. The supernatant was then loaded onto an SP Sepharose Fast Flow column (Cytiva, Marlborough, MA) and washed with lysis buffer, and bound proteins were eluted using a 50–300 mM NaCl gradient. Ub was eluted into 100–150 mM NaCl fractions and further purified using size exclusion chromatography using a HiLoad 16/60 Superdex 75 prep grade column (Cytiva). The elution fraction was stored at −30°C until use in NMR experiments.

THz-HDX experiments

A stock solution of Ub in 50 mM sodium phosphate buffer (pH 6.4; also see main text for other pH values tested) containing 50 mM NaCl was diluted to 0.2 mM with the same buffer. Then, 250 μL of Ub solution was added to 1.5-mL tubes and flash frozen in liquid nitrogen, followed by lyophilization in a freeze-dryer FDU-2100 (EYELA, Tokyo, Japan) at room temperature and under a pressure of ~5.5 Pa for 36 h. This treatment eliminates bulk water while preserving bound water on the protein surface (48,49).

The HDX experiment was initiated by dissolving lyophilized Ub in 100 μL D_2O to prepare a 0.5-mM Ub solution at room temperature (Fig. 1 *C*). The D_2O solution of Ub was immediately transferred into the sample cell, and subterahertz irradiation was initiated 1 min after dissolution and was continued for 3, 6, or 12 min. After irradiation, the Ub solution was collected in a 1.5-mL tube and stored at 4°C to minimize subsequent HDX (Fig. 1 *C*). For all samples, the time from D_2O addition at room temperature to storage at 4°C was fixed at 14 min. Temperature control (TC) experimental samples were prepared for each time point by inserting a water layer between the Ub solution and subterahertz source to shield the Ub solution from direct irradiation while allowing passive temperature rise via thermal conduction from the subterahertz-heated water (Fig. 1 *B*). In addition, general control (GC) experimental samples not subjected to subterahertz irradiation or temperature elevation but only incubated in the sample cell were prepared.

All samples were mixed with 150 μL of ice-cold D_2O-based buffer solution with the same composition as the ubiquitin stock buffer and then transferred into prechilled microtubes with an outer diameter of 5 mm (Shigemi, Tokyo, Japan) at 4°C. NMR experiments were performed using a Bruker Avance III 800 MHz spectrometer equipped with a TXO triple-resonance cryoprobe (Bruker, Billerica, MA). The samples were loaded onto a magnet set at 4°C under nitrogen gas flow. After frequency lock, tuning and matching of radiofrequency coils, and shimming, three ^{1}H-^{15}N SOFAST-HMQC spectra were sequentially acquired at 4°C (50). Each of the three spectra were acquired in 5 min 55 s, with acquisition times in the direct and indirect dimensions of 45.9 ms (*t*2, ^{1}H) and 43.2 ms (*t*1, ^{15}N), spectral widths of 14 and 32 ppm, number of scans of four per indirect *t*1 point, and with a interscan delay of 0.3 s. Three spectra were summed (~18 min in total) to enhance S/N, as we verified the absence of progress of HDX during the measurement. We observed no significant differences in the average signal intensities among the three spectra (overall, the standard deviation was 2–3% of the mean). After the measurement, samples were incubated at room temperature for >12 h to equilibrate HDX, followed by a ^{1}H-^{13}C constant-time HSQC experiment (51).

All the experiments were repeated two or more times, and representative results are shown. The HDX and NMR experiments were performed at the AIST Tsukuba Central and AIST Tokyo Waterfront, respectively. All samples were kept at 4°C for ~2 h of transportation between the two institutions.

NMR spectroscopic data analyses

Time-domain data were multiplied by Gaussian and π/2-shifted squared sine window functions on *t*2 free induction decays and *t*1 interferograms, respectively, followed by Fourier transformation and phase and baseline corrections in the TopSpin 2.1 software (Bruker). The spectra were analyzed using Sparky (52). To account for the effects of subtle differences in Ub concentrations among samples, amide signal intensities were normalized using signals from unexchangeable groups as internal controls. For this purpose, the heights of 37 methyl resonances in the ^{1}H-^{13}C constant-time HSQC spectra were used (Fig. S1). The ratio of subterahertz intensity (or TC) to that of GC was used to evaluate the effects of subterahertz irradiation (or temperature elevation) on HDX.

$$R_t = \frac{I_{i,THz\ (or\ TC),\ t}}{I_{i,\ GC,t}}, \quad (1)$$

where $t = 3$, 6, and 12 min. Under subterahertz wave- or heat-induced HDX acceleration, this ratio is <1, whereas under deceleration, it is >1. However, because the temperature elevation induced by subterahertz irradiation equilibrates after ~3 min, we used differences in R_t at 6 and 12 min from $R_{3\ \mathrm{min}}$ as quantitative criteria for deciding whether the HDX is accelerated or decelerated.

$$\Delta R = R_t - R_{3\ \mathrm{min}}. \quad (2)$$

If HDX is accelerated (or decelerated) by subterahertz irradiation or heating after temperature equilibration, ΔR will be negative (or positive) to an extent larger than its error,

$$\Delta R + Error(\Delta R) < 0 \rightarrow \text{acceleration}, \quad (3)$$

$$\Delta R - Error(\Delta R) > 0 \rightarrow \text{deceleration}. \quad (4)$$

$Error(\Delta R)$ was defined using the sum of each *Error* at two time points *t* and 3 min as follows:

$$Error(\Delta R) = [Error(R_t) + Error(R_{3\ \mathrm{min}})]/\sqrt{2}, \quad (5)$$

where each $Error(R_t)$ was estimated from the signal/noise ratio (SNR) considering error propagation according to (53):

$$Error(R_t) = R_t\sqrt{\left(1/SNR_{THz\ (TC),\ t}\right)^2 + \left(1/SNR_{GC,\ t}\right)^2}. \quad (6)$$

RESULTS

We monitored the main-chain amide protons of Ub as they were being exchanged with solvent deuterons after the dissolution of the lyophilized Ub in D_2O. In general, lyophilization eliminates bulk H_2O from protein solutions while preserving bound H_2O on protein surfaces (48,49). Although lyophilization could induce protein unfolding or structural collapse (54), no such effect was observed in the Ub used in our experiment (Supporting materials and methods; Fig. S3). For subterahertz excitation, we applied intense 0.1-THz electromagnetic pulses with high (90 mW/cm^2) or low (18 mW/cm^2) power density to the Ub solution by constructing an optical setup using subterahertz klystron (Fig. 1 *A*). We measured the elevation in the volume-averaged temperature for the subterahertz-irradiated sample by immersing a thermocouple in the sample solution. The temperature gradually increased for 3 min, and the plateau was estimated to be 5 and 0.3°C for the high- and low-power density irradiations, respectively. For the TC experiment, we similarly raised the sample temperature by 5°C through a water layer attached to the subterahertz-exposed surface of the SiO_2 window (Fig. 1 *B*). This layer completely absorbed the subterahertz radiation and allowed the heat transfer to the sample in a beam-power-controlled manner without direct subterahertz irradiation. After irradiation or heating for 3, 6, or 12 min, all samples were further incubated at room temperature to ensure a constant duration of 14 min after dissolving in D_2O (Fig. 1 *C*). The Ub solution was then stored for 6–10 h at 4°C to minimize the subsequent

exchange until NMR measurement. For the GC experiment, the same experiment using the same subterahertz-klystron setup was conducted by cutting off the terahertz radiation.

We obtained two-dimensional amide ^{1}H-^{15}N SOFAST-HMQC spectra of Ub after exposure to 90- or 18-mW/cm^2 subterahertz radiation. In these spectra, resonances of non-proline amino acid residues were observed at distinct chemical shifts, whose intensities reflect the extent of HDX, as the deuterated amide moieties after HDX were not detected in the experiment. The spectra of the amino acid residues were compared with those of TC and GC. We verified that there was no significant difference in the spectral patterns with and without subterahertz irradiation, indicating that the applied intense 0.1-THz pulses did not cause irreversible changes (e.g., denaturation, oxidation, deamination, and isomerization) to the protein structure (Fig. S4). The target residues of interest were selected by excluding those with 1) fast HDX rates, that is, being exchanged during the sample storage time at 4°C within 6–10 h (Fig. S2); 2) relatively low signal intensity (signal/noise ratio of <33.3, which corresponds to >3% noise level relative to the intensity); and 3) overlapping of two signals.

For the selected 21 residues, we estimated the residue-specific HDX rate for the GC sample based on real-time NMR measurement, which showed that HDX rate of these residues are smaller than ~0.1 h^{-1} (Fig. S5). It should be noted that the signal intensity of several residues was increased time dependently, supposedly because of reduced dipolar broadening (55). However, this should not substantially affect the interpretation of THz-HDX data, as the change would have been equilibrated within the delay of 6–10 h before NMR measurements (Fig. S2).

Next, the selected 21 residues classified as significantly accelerated or decelerated after subterahertz irradiation or heating (Fig. 2, *inset*) were mapped onto the Ub structure (Fig. 2; Fig. S6). We observed that irradiation at 18 mW/cm^2 accelerated HDX in the residues located in the inner helical surface (Fig. 2, *left*; V26 and I30) and the hydrophobic surface composed of four β-strands, $\beta1/\beta2$ and $\beta3/\beta5$ (Fig. 2, *left*; I3, F4, L15, V17, I44, and V70), whereas it decelerated HDX in residues located in the surface loop and 3_{10} helix (Fig. 2, *left*; D21, Y59, and I61). Notably, these effects were qualitatively contradictory to those of temperature rise by 5°C; thus, the inner helical and hydrophobic patch regions were characterized by decelerated HDX, and the surface loop and 3_{10} helix regions were characterized by accelerated HDX (Fig. 2, *right*). Moreover, irradiation at 90 mW/cm^2 with the same 5°C rise in temperature appeared to counteract the subterahertz-radiation-induced effects because the entire profile of HDX changes occurred approximately in the middle or mixture of these two effects (Fig. 2, *middle*). Therefore, it is unlikely that the temperature increase induced by subterahertz irradiation altered Ub dynamics. Alternatively, subterahertz energy could have directly excited the hydrogen-bond networks (i.e., intermolecular motions) of the protein and surrounding water molecules with various microscopic properties.

The subterahertz-radiation- and heat-induced effects observed in the HDX profiles may be overestimated because of the variation in time intervals between the subterahertz (or heat) perturbation and NMR probing of each sample (Fig. S2). This variation can affect the extent of HDX, depending on the rate. However, it was verified by the experimentally determined HDX rates for the GC sample that the history of the subterahertz-radiation- or heat-induced effect was reasonably maintained in their HDX profiles (Fig. S5). Moreover, in several residues with relatively fast HDX rates, the opposite effects observed between subterahertz irradiation and heating were underestimated by the variation in the perturbing and probing intervals (Fig. S7).

To determine whether the 6–10 h period of storage at 4°C (because of the long physical distance between the subterahertz-klystron and NMR devices; see Materials and methods) after irradiation could produce artifacts in the HDX data, we performed another THz-HDX experiment with an IMPATT-diode-based 0.1-THz light source installed near the NMR spectrometer. This experimental setup allowed us to substantially shorten the time interval to 12 min at room temperature (Fig. S8 *A*). The results showed that this subterahertz irradiation for 3 min without long-term storage caused an effect similar to that of klystron-based irradiation, i.e., an effect opposite to that of the temperature rise in the HDX profile (Fig. S9). Details of the results and experimental setup are shown in the Supporting materials and methods and Fig. S8.

DISCUSSION

We interpreted the influence of subterahertz irradiation on HDX kinetics in Ub as a type of solvent effect, which depends on its structure and the surrounding hydrogen-bond networks. This interpretation is supported by the strong pH dependence of the HDX rate and lack of partial denaturation of Ub during lyophilization (Supporting materials and methods; Figs. S3 and S10). Their experimental verifications are essential to explain the phenomena in line with the framework of a well-established kinetic model of HDX; protonated backbone amides (NH) involved in hydrogen-bonded secondary or tertiary structures or sequestered within the protein can be exchanged with the solvent deuterons in an open state to the solvent (37,56), as indicated by the kinetic scheme described by Englander's group (57).

$$NH(closed) \overset{K_{op}}{\rightleftharpoons} NH(open) \overset{k_{ch}}{\rightarrow} exchanged. \qquad (7)$$

In typical HDX experiments for native proteins, the rate-limiting step is mostly observed during chemical exchange, and structural opening and closing are regarded as pre-equilibrium. This is known as the EX2 limit, and it is distinct from the alternative EX1 limit at which the HDX rate is

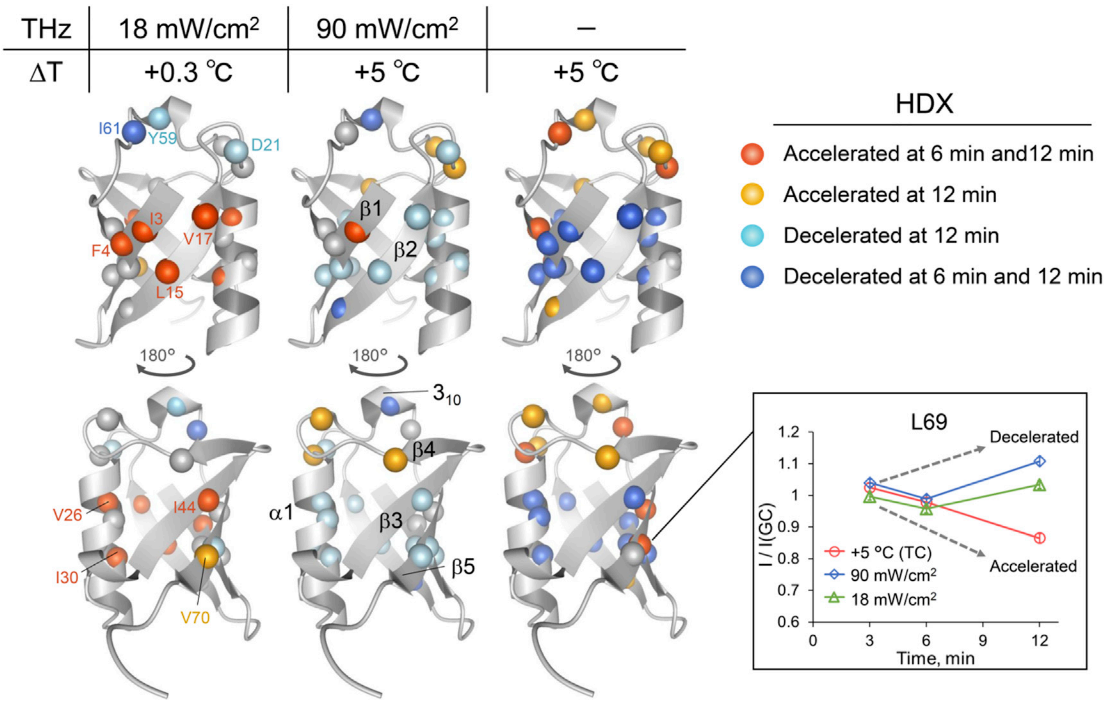

FIGURE 2 Effect of subterahertz irradiation at low (*left*) and high (*middle*) power density and temperature increase (*right*) on the amide proton exchange of Ub. The tertiary Ub structures with 180° rotation are shown (Protein Data Bank, PDB: 1UBQ) (42). Amide nitrogen atoms of the analyzed residues are shown (see main text for details). Amino acid residues were mapped in the Ub structure when the HDX of the main chain amide groups was accelerated or decelerated. An example of L69 is shown in an inset. The acceleration or deceleration of HDX in each residue (schematically indicated with an *arrow*) was defined using the signal intensity ratio of each measurement to that of GC ($I/I_{(GC)}$; see Materials and methods for details). When $I/I_{(GC)}$ was decreased or increased (i.e., HDX was accelerated or decelerated) after subterahertz radiation or heating above the measurement error range, the corresponding residue was colored orange or blue, respectively. Dark and light colors indicate that the acceleration or deceleration of HDX was detected at 6 and 12 min and only at 12 min, respectively. Note that 3 min was selected as the reference time point at which the sample temperature reached plateau after subterahertz irradiation or heating. Error bars are derived from signal/noise ratio, following Eq. 6.

determined by the opening rate (58). In the EX2 limit, the exchange rate constant of any hydrogen (k_{ex}) is determined by its chemical exchange rate constant in the open form (k_{ch}) multiplied by the equilibrium opening constant (K_{op}), as follows (57):

$$k_{ex} = K_{op} k_{ch}. \quad (8)$$

In our measurement, the HDX rates of the selected 21 Ub residues were strongly affected after a pH change from 5.6 to 7.2 (Fig. S10), consistent with the view that the HDX rates of these residues generally follow EX2 behavior (59). However, the slow HDX residues primarily located in the protein interior and the hydrophobic surface (e.g., Q41, R42, and L43 located in β3) were much less sensitive to the pH change than a 10-fold increase per pH unit expected for an ideal EX2 exchange rate (58). Such structural regions are rigid and less frequently open owing to intramolecular hydrogen-bond networks that are found between two β strands and hydrophobic interactions (30). This suggests that in these residues, protein folding (opening constant, K_{op}) significantly affected the HDX rates (k_{ex}).

Our NMR measurements showed that in the protein interior and hydrophobic surface (hydrophobic regions), HDX decelerated as the temperature increased from 25 to 30°C (Fig. 2, *right*). This indicates that the temperature rise by 5°C could optimize the conformation that makes the hydrogen-bond network surrounding the structural regions more rigid (smaller K_{op}), thereby reducing the accessibility of the local solvent, D_2O, to the open form of the protein. In fact, some residues in the β-sheet of Ub, including I44 and V70, may be associated with cold denaturation, whose backbone hydrogen bonds become longer under supercooled conditions (60). Conversely, HDX of the surface loop and short 3_{10} helix regions (hydrophilic regions) became rapid with the temperature rise. This is likely because the chemical exchange rate is little affected by the protein folding ($k_{ex} \approx k_{ch}$) and thus is increased by the temperature rise as expected only from the activation energy determining k_{ch} (59).

The opposite effect of the temperature rise on HDX kinetics was observed with 0.1-THz irradiation. This can be explained by a solvent effect that can lead to the following: 1) a larger K_{op} for the hydrophobic regions and 2) a smaller k_{ch} for the hydrophilic regions where $k_{ex} \approx k_{ch}$ is expected. The hydrophobic regions will be structurally unstable (larger

K_{op}) when subterahertz radiation induces the recombination of intramolecular hydrogen bonds with the intermolecular bonds in the presence of the solvent D_2O. In contrast, the hydrophilic regions would be dominated by intermolecular hydrogen bonds with the solvent even before subterahertz irradiation. In fact, bound or slow water has been detected in the regions by solution NMR studies using reverse micelle encapsulation (39) and by molecular dynamics simulations (40). Thus, k_{ch} would be smaller if the subterahertz excitation blocked the access of the solvent D_2O to the protonated backbone amides for the exchange via inducing a more extended hydrogen-bond network surrounding the hydrophilic regions. Although we could not determine the underlying mechanism, the number of recombination trials of the hydrogen-bond network required for its extension will be significantly increased on the 10-ps timescale by the subterahertz excitation. In this scenario, it may not be necessary to assume an extremely long residence time of a particular bound water, which contradicts previous views on Ub dynamics (61).

CONCLUSION

To date, limited information is available regarding whether an externally applied alternating electromagnetic field with subterahertz frequency can directly and nonthermally influence protein and hydration dynamics, thus affecting biological functions. In this study, we investigated the difference between subterahertz-radiation- and heat-induced effects on the dynamics of Ub by developing a THz-HDX approach that combines klystron-based 0.1-THz irradiation with NMR-based HDX measurement. Using this method, we demonstrated that 0.1-THz irradiation affected Ub dynamics in a manner reflecting the heterogeneous nature of the hydrogen-bond network around the protein. Interestingly, the effect observed after 0.1-THz irradiation was opposite to that observed after temperature increase, suggesting that the applied 0.1-THz radiation energy was retained in specific protein and water interactions. Our results will help to expand our understanding of the relationship between the microscopic properties of fast-fluctuating dynamics and slow biological functions. Future studies should focus on elucidating the mechanism through which 0.1-THz excitation at the 10-ps timescale can influence the protein and hydration interactions of much slower time domains and cause an effect opposite to heating.

SUPPORTING MATERIAL

Supporting material can be found online at https://doi.org/10.1016/j.bpj.2021.04.013.

AUTHOR CONTRIBUTIONS

M.I., Y. Tokunaga, K.T., and M.T. designed the research. Y. Tokunaga, M.I., M.T., H.I., M.K., Y. Tojima, and K.T. performed the research. Y. Tokunaga analyzed the data. M.I. and Y. Tokunaga interpreted the data and wrote the manuscript, which was edited by all authors.

ACKNOWLEDGMENTS

We thank Hiromichi Hoshina, Hiroshi Murakami, and David B. Lukatsky for the critical reading of the manuscript and Satoshi Takahashi and Nobuo Shimamoto for insightful discussions. We also thank Editage for English language editing.

This work was funded by JSPS KAKENHI (grant numbers 18K18731 and 20H03298) and by the Research Foundation for Opto-Science and Technology (to M.I.). This work is based on the results obtained with the support of the RIKEN–AIST Joint Research Fund (Semi-full research).

REFERENCES

1. Schirò, G., and M. Weik. 2019. Role of hydration water in the onset of protein structural dynamics. *J. Phys. Condens. Matter.* 31:463002.
2. Laage, D., T. Elsaesser, and J. T. Hynes. 2017. Water dynamics in the hydration shells of biomolecules. *Chem. Rev.* 117:10694–10725.
3. Rupley, J. A., and G. Careri. 1991. Protein hydration and function. *Adv. Protein Chem.* 41:37–172.
4. Bellissent-Funel, M. C., A. Hassanali, ..., A. E. Garcia. 2016. Water determines the structure and dynamics of proteins. *Chem. Rev.* 116:7673–7697.
5. Biedermannová, L., and B. Schneider. 2016. Hydration of proteins and nucleic acids: advances in experiment and theory. A review. *Biochim. Biophys. Acta.* 1860:1821–1835.
6. Khodadadi, S., and A. P. Sokolov. 2017. Atomistic details of protein dynamics and the role of hydration water. *Biochim. Biophys. Acta Gen. Subj.* 1861:3546–3552, Published online May 4, 2016.
7. Khodadadi, S., J. H. Roh, ..., A. P. Sokolov. 2010. Dynamics of biological macromolecules: not a simple slaving by hydration water. *Biophys. J.* 98:1321–1326.
8. Yamamoto, N., S. Ito, ..., K. Tominaga. 2018. Effect of temperature and hydration level on purple membrane dynamics studied using broadband dielectric spectroscopy from sub-GHz to THz regions. *J. Phys. Chem. B.* 122:1367–1377.
9. Wheaton, S., R. Gelfand, and R. Gordon. 2015. Probing the Raman-active acoustic vibrations of nanoparticles with extraordinary spectral resolution. *Nat. Photonics.* 9:68–72.
10. He, Y., J. Y. Chen, ..., A. G. Markelz. 2011. Evidence of protein collective motions on the picosecond timescale. *Biophys. J.* 100:1058–1065.
11. Lipps, F., S. Levy, and A. G. Markelz. 2012. Hydration and temperature interdependence of protein picosecond dynamics. *Phys. Chem. Chem. Phys.* 14:6375–6381.
12. Marques, B. S., M. A. Stetz, ..., N. V. Nucci. 2020. Protein conformational entropy is not slaved to water. *Sci. Rep.* 10:17587.
13. Brooks, B., and M. Karplus. 1985. Normal modes for specific motions of macromolecules: application to the hinge-bending mode of lysozyme. *Proc. Natl. Acad. Sci. USA.* 82:4995–4999.
14. Shiraga, K., Y. Ogawa, and N. Kondo. 2016. Hydrogen bond network of water around protein investigated with terahertz and infrared spectroscopy. *Biophys. J.* 111:2629–2641.
15. Yamamoto, N., K. Ohta, ..., K. Tominaga. 2016. Broadband dielectric spectroscopy on lysozyme in the sub-gigahertz to terahertz frequency regions: effects of hydration and thermal excitation. *J. Phys. Chem. B.* 120:4743–4755.
16. Kabir, S. R., K. Yokoyama, ..., M. Suzuki. 2003. Hyper-mobile water is induced around actin filaments. *Biophys. J.* 85:3154–3161.
17. Shiraga, K., K. Tanaka, ..., Y. Ogawa. 2018. Reconsideration of the relaxational and vibrational line shapes of liquid water based on ultrabroadband dielectric spectroscopy. *Phys. Chem. Chem. Phys.* 20:26200–26209.

18. Niessen, K. A., M. Xu, …, A. G. Markelz. 2019. Protein and RNA dynamical fingerprinting. *Nat. Commun.* 10:1026.
19. Heyden, M., and M. Havenith. 2010. Combining THz spectroscopy and MD simulations to study protein-hydration coupling. *Methods.* 52:74–83.
20. Xu, Y., and M. Havenith. 2015. Perspective: watching low-frequency vibrations of water in biomolecular recognition by THz spectroscopy. *J. Chem. Phys.* 143:170901.
21. Turton, D. A., H. M. Senn, …, K. Wynne. 2014. Terahertz underdamped vibrational motion governs protein-ligand binding in solution. *Nat. Commun.* 5:3999.
22. Dielmann-Gessner, J., M. Grossman, …, I. Sagi. 2014. Enzymatic turnover of macromolecules generates long-lasting protein-water-coupled motions beyond reaction steady state. *Proc. Natl. Acad. Sci. USA.* 111:17857–17862.
23. González-Jiménez, M., G. Ramakrishnan, …, K. Wynne. 2016. Observation of coherent delocalized phonon-like modes in DNA under physiological conditions. *Nat. Commun.* 7:11799.
24. Conti Nibali, V., and M. Havenith. 2014. New insights into the role of water in biological function: studying solvated biomolecules using terahertz absorption spectroscopy in conjunction with molecular dynamics simulations. *J. Am. Chem. Soc.* 136:12800–12807.
25. Greschner, A. A., X. Ropagnol, …, M. A. Gauthier. 2019. Room-temperature and selective triggering of supramolecular DNA assembly/disassembly by nonionizing radiation. *J. Am. Chem. Soc.* 141:3456–3469.
26. Lundholm, I. V., H. Rodilla, …, G. Katona. 2015. Terahertz radiation induces non-thermal structural changes associated with Fröhlich condensation in a protein crystal. *Struct. Dyn.* 2:054702.
27. Yamazaki, S., M. Harata, …, Y. Ogawa. 2018. Actin polymerization is activated by terahertz irradiation. *Sci. Rep.* 8:9990.
28. Serdyukov, D. S., T. N. Goryachkovskaya, …, S. E. Peltek. 2020. Study on the effects of terahertz radiation on gene networks of *Escherichia coli* by means of fluorescent biosensors. *Biomed. Opt. Express.* 11:5258–5273.
29. Kitazawa, S., Y. Aoshima, …, R. Kitahara. 2018. Water-protein interactions coupled with protein conformational transition. *Biophys. J.* 115:981–987.
30. Haririnia, A., R. Verma, …, D. Fushman. 2008. Mutations in the hydrophobic core of ubiquitin differentially affect its recognition by receptor proteins. *J. Mol. Biol.* 375:979–996.
31. Krishna, M. M., L. Hoang, …, S. W. Englander. 2004. Hydrogen exchange methods to study protein folding. *Methods.* 34:51–64.
32. Woodward, C., N. Carulla, and G. Barany. 2004. Native state hydrogen-exchange analysis of protein folding and protein motional domains. *Methods Enzymol.* 380:379–400.
33. Goldstein, G., M. Scheid, …, E. A. Boyse. 1975. Isolation of a polypeptide that has lymphocyte-differentiating properties and is probably represented universally in living cells. *Proc. Natl. Acad. Sci. USA.* 72:11–15.
34. Ibarra-Molero, B., V. V. Loladze, …, J. M. Sanchez-Ruiz. 1999. Thermal versus guanidine-induced unfolding of ubiquitin. An analysis in terms of the contributions from charge-charge interactions to protein stability. *Biochemistry.* 38:8138–8149.
35. Johnson, E. C., G. A. Lazar, …, T. M. Handel. 1999. Solution structure and dynamics of a designed hydrophobic core variant of ubiquitin. *Structure.* 7:967–976.
36. Kitazawa, S., M. Yagi-Utsumi, …, R. Kitahara. 2017. Interactions controlling the slow dynamic conformational motions of ubiquitin. *Molecules.* 22:E1414.
37. Pan, Y., and M. S. Briggs. 1992. Hydrogen exchange in native and alcohol forms of ubiquitin. *Biochemistry.* 31:11405–11412.
38. Chandak, M. S., T. Nakamura, …, K. Kuwajima. 2013. The use of spin desalting columns in DMSO-quenched H/D-exchange NMR experiments. *Protein Sci.* 22:486–491.
39. Nucci, N. V., M. S. Pometun, and A. J. Wand. 2011. Site-resolved measurement of water-protein interactions by solution NMR. *Nat. Struct. Mol. Biol.* 18:245–249.
40. Duboué-Dijon, E., and D. Laage. 2014. Comparative study of hydration shell dynamics around a hyperactive antifreeze protein and around ubiquitin. *J. Chem. Phys.* 141:22D529.
41. Heyden, M. 2019. Heterogeneity of water structure and dynamics at the protein-water interface. *J. Chem. Phys.* 150:094701.
42. Vijay-Kumar, S., C. E. Bugg, and W. J. Cook. 1987. Structure of ubiquitin refined at 1.8 A resolution. *J. Mol. Biol.* 194:531–544.
43. Born, B., S. J. Kim, …, M. Havenith. 2009. The terahertz dance of water with the proteins: the effect of protein flexibility on the dynamical hydration shell of ubiquitin. *Faraday Discuss.* 141:161–173, discussion 175–207..
44. Wirtz, H., S. Schäfer, …, M. Havenith. 2018. Hydrophobic collapse of ubiquitin generates rapid protein-water motions. *Biochemistry.* 57:3650–3657.
45. Manna, B., A. Nandi, …, D. K. Palit. 2020. Effect of aggregation on hydration of HSA protein: steady-state Terahertz absorption spectroscopic study. *J. Chem. Sci.* 132:8.
46. Iida, H., M. Kinoshita, and K. Amemiya. 2018. Accurate measurement of absolute terahertz power using broadband calorimeter. *Int. J. Infrared Millim. Terahertz Waves.* 39:409–421.
47. Moriya, J., M. Sakakura, …, I. Shimada. 2009. An NMR method for the determination of protein binding interfaces using TEMPOL-induced chemical shift perturbations. *Biochim. Biophys. Acta.* 1790:1368–1376.
48. Lim, J. Y., N. A. Kim, …, S. H. Jeong. 2016. Process cycle development of freeze drying for therapeutic proteins with stability evaluation. *J. Pharm. Investig.* 46:519–536.
49. Nireesha, G. R., L. Divya, …, V. Lavakumar. 2013. Lyophilization/freeze drying - an review. *Int. J. Novel Trends Pharm. Sci.* 3:87–98.
50. Schanda, P., E. Kupce, and B. Brutscher. 2005. SOFAST-HMQC experiments for recording two-dimensional heteronuclear correlation spectra of proteins within a few seconds. *J. Biomol. NMR.* 33:199–211.
51. Vuister, W. G., and A. Bax. 1992. Resolution enhancement and spectral editing of uniformly 1^{3C}-enriched proteins by homonuclear broadband 1^{3C} decoupling. *J. Magn. Reson.* 98:428–435.
52. Lee, W., M. Tonelli, and J. L. Markley. 2015. NMRFAM-SPARKY: enhanced software for biomolecular NMR spectroscopy. *Bioinformatics.* 31:1325–1327.
53. Wakamoto, T., T. Ikeya, …, R. Kitahara. 2019. Paramagnetic relaxation enhancement-assisted structural characterization of a partially disordered conformation of ubiquitin. *Protein Sci.* 28:1993–2003.
54. Desai, U., J. Osterhout, and A. Klibanov. 1994. Protein-structure in the lyophilized state - a hydrogen isotope-exchange NMR-study with bovine pancreatic trypsin-inhibitor. *J. Am. Chem. Soc.* 116:9420–9422.
55. Gledhill, J. M., Jr., B. T. Walters, and A. J. Wand. 2009. AMORE-HX: a multidimensional optimization of radial enhanced NMR-sampled hydrogen exchange. *J. Biomol. NMR.* 45:233–239.
56. Woodward, C., I. Simon, and E. Tüchsen. 1982. Hydrogen exchange and the dynamic structure of proteins. *Mol. Cell. Biochem.* 48:135–160.
57. Bai, Y., T. R. Sosnick, …, S. W. Englander. 1995. Protein folding intermediates: native-state hydrogen exchange. *Science.* 269:192–197.
58. Englander, S. W., T. R. Sosnick, …, L. Mayne. 1996. Mechanisms and uses of hydrogen exchange. *Curr. Opin. Struct. Biol.* 6:18–23.
59. Bai, Y., J. S. Milne, …, S. W. Englander. 1993. Primary structure effects on peptide group hydrogen exchange. *Proteins.* 17:75–86.
60. Cho, M. K., S. Xiang, …, M. Zweckstetter. 2012. Cold-induced changes in the protein ubiquitin. *PLoS One.* 7:e37270.
61. Persson, E., and B. Halle. 2008. Nanosecond to microsecond protein dynamics probed by magnetic relaxation dispersion of buried water molecules. *J. Am. Chem. Soc.* 130:1774–1787.

Biophysical Journal, Volume *120*

Supplemental information

Nonthermal excitation effects mediated by sub-terahertz radiation on hydrogen exchange in ubiquitin

Yuji Tokunaga, Masahito Tanaka, Hitoshi Iida, Moto Kinoshita, Yuya Tojima, Koh Takeuchi, and Masahiko Imashimizu

Supplementary Text

The effect of lyophilization on the native Ub structure.

In addition to lyophilization-dissolution (LD), real-time HDX experiments were also initiated by buffer exchange via dilution, followed by concentration with ultrafiltration (UF). Although lyophilization could induce protein unfolding or structural collapse (54), we selected the LD procedure because of the following reasons: (i) LD requires a shorter time before NMR measurement and thus enhances reproducibility, and (ii) Ub is a highly stable protein. We compared the HDX profiles of Ub initiated by LD and UF procedures, which showed overlapping chemical shift patterns in the two spectra (Supplementary Fig. S3A). Moreover, the rate constants of the eight residues that could be determined under the experimental condition were nearly identical between the two experiments (Supplementary Fig. S3B). Therefore, we concluded that no denaturing effect occurred during lyophilization.

Method of THz-HDX experiment using an IMPATT diode-based light source

Procedures for preparation of lyophilized Ub, initiation of HDX by dissolving in D_2O, sub-THz irradiation, transfer to an NMR tube, and acquiring and processing NMR data are the same as those described for the THz-HDX experiments using the klystron-based light source, except followings: (i) Duration at room temperature was prolonged from 14 min to 62 min, in which sub-THz was irradiated for 3 min or 60 min. (ii) A continuous-wave IMPATT diode (Terasense, San Jose, CA) was used as a portable 0.1 THz light source (see the legend of Supplementary Fig. S8 for the details). This source can generate 96.9 GHz radiation and 181 mW power, which was pulse-modulated at a 50-Hz repetition rate, and a 20-ms pulse width of a square wave. The power density of the 0.1-THz radiation transmitted to the sample surface was measured as described in Materials and Methods, which was estimated to be 24 mW/cm^2. (iii) Delay of NMR measurement after sub-THz irradiation was shortened from 6-10 h to 12 min. This was made possible by using the portable IMPATT diode-based light source in AIST Tokyo Waterfront, where the NMR spectrometer is placed.

Result of THz-HDX experiment using an IMPATT diode-based light source

We investigated whether the 6-10 h delay of NMR measurement after sub-THz irradiation can significantly affect our interpretation of the sub-THz-induced effect on HDX kinetics. In order to shorten the delay, we constructed another experimental setup for THz-HDX system with a portable 0.1 THz light source (IMPATT diode device) that was installed near the NMR spectrometer. Using the IMPATT diode device, we applied 0.1-THz radiation with a power density of 24 mW/cm^2 to the Ub solution for 3 min or 60 min, in the same manner as the klystron-based experiment (Supplementary Fig. S8A), except that (i) the delay of NMR

measurement was 12 min and (ii) buffer pH was decreased from 6.4 to 5.6. The acidic pH generally decreases the chemical exchange rate (57-59), allowing a prolonged sub-THz irradiation during HDX at room temperature. HDX reactions were then measured by NMR after a total of 74 min of Ub dissolution in D_2O (Supplementary Fig. S8A).

As similarly observed in klystron-based irradiation at 18 mW/cm^2, the IMPATT-diode-based irradiation at 24 mW/cm^2 for 3 min induced changes in HDX kinetics opposite to those induced by increased temperature (compare Supplementary Fig. S8 B and C with Fig. 2). Notably, the irradiated HDX profiles negatively correlates with the non-irradiated profile with 5 °C temperature rise (TC), irrespective of the types of the sub-THz-sources and the delay of NMR measurement (Supplementary Fig. S9). In contrast, when the irradiation duration was increased to 60 min, the HDX profile of IMPATT-diode-based irradiation was similar to (i.e., positively correlates with) that of TC (Supplementary Fig. S9). The IMPATT-diode-based irradiation for 3 min and 60 min increased volume-averaged sample temperatures by 0.5 °C and 2 °C, respectively, with a plateau of approximately 20 min. The relatively enhanced temperature rise per power density compared to the klystron-based irradiation is due to its much longer pulse duration (i.e. less likely thermal diffusion per duty cycle). Therefore, the sub-THz-induced nonthermal effect could be dominated by the effect of heat dissipated via the prolonged irradiation for 60 min. This result is also consistent with the fact that the high-power klystron-based irradiation at 90 mW/cm^2, which increased the sample temperature by 5 °C, made the nonthermal effect on the HDX profile closer to the heating effect (Fig. 2).

Supplementary Figures

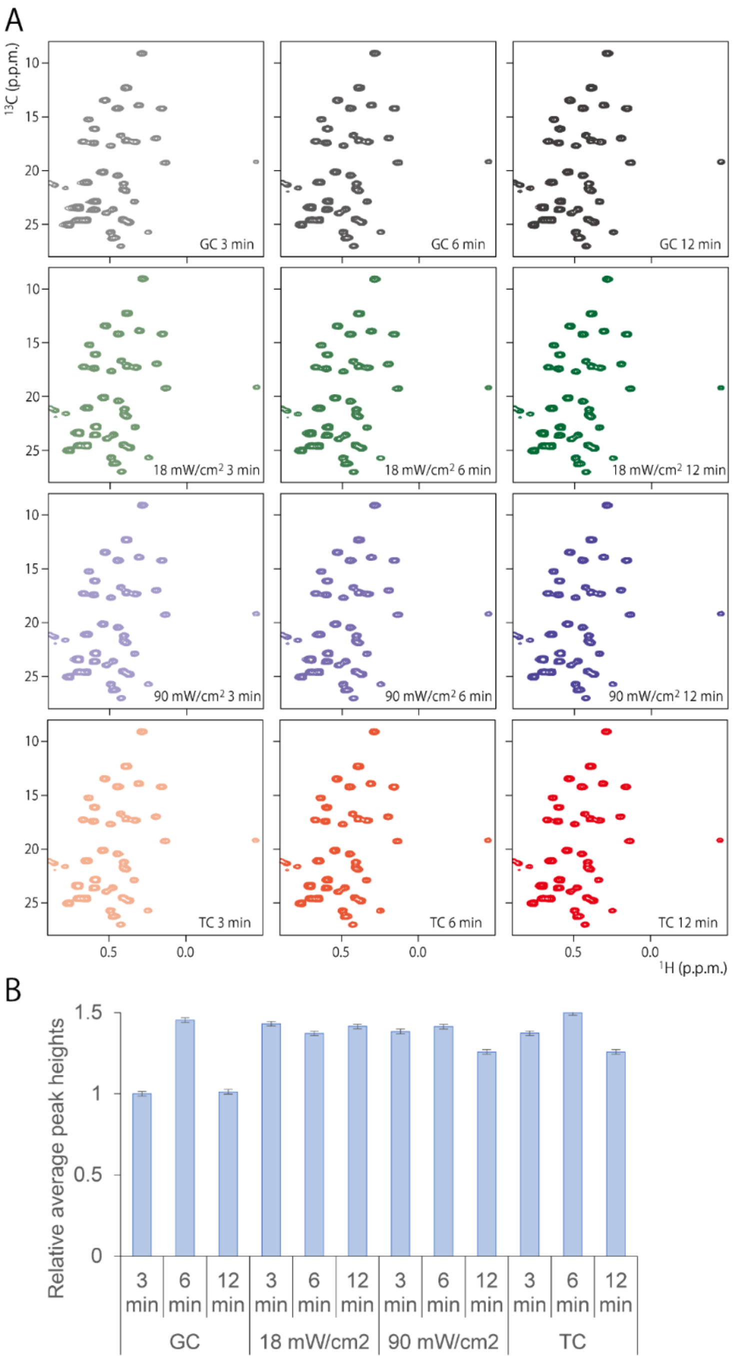

Supplementary Fig. S1. Normalization of amide signal peak heights using those from unexchangeable proton sites. (**A**) ^{1}H-^{13}C constant-time HSQC spectra of THz-HDX samples. Peak heights of 37 methyl resonances were analyzed. (**B**) Relative average peak height of 37 methyl resonances of each sample, in which GC-3 min is set to 1. Error bars are derived from signal-to-noise ratios. Assuming that these values reflect the differences in Ub concentration among samples, peak heights of amide signals were divided by these values to exclude the effect of such differences.

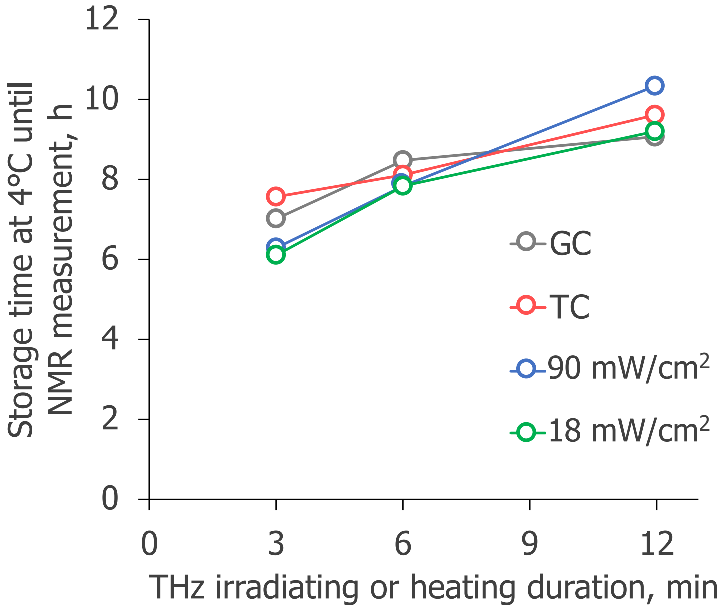

Supplementary Fig. S2. Variation in storage times at 4 °C until NMR measurement (i.e., variation in time intervals between the THz/heat perturbations and NMR probing) of each sample. GC and TC represent the general control experiment and the temperature control experiment, respectively.

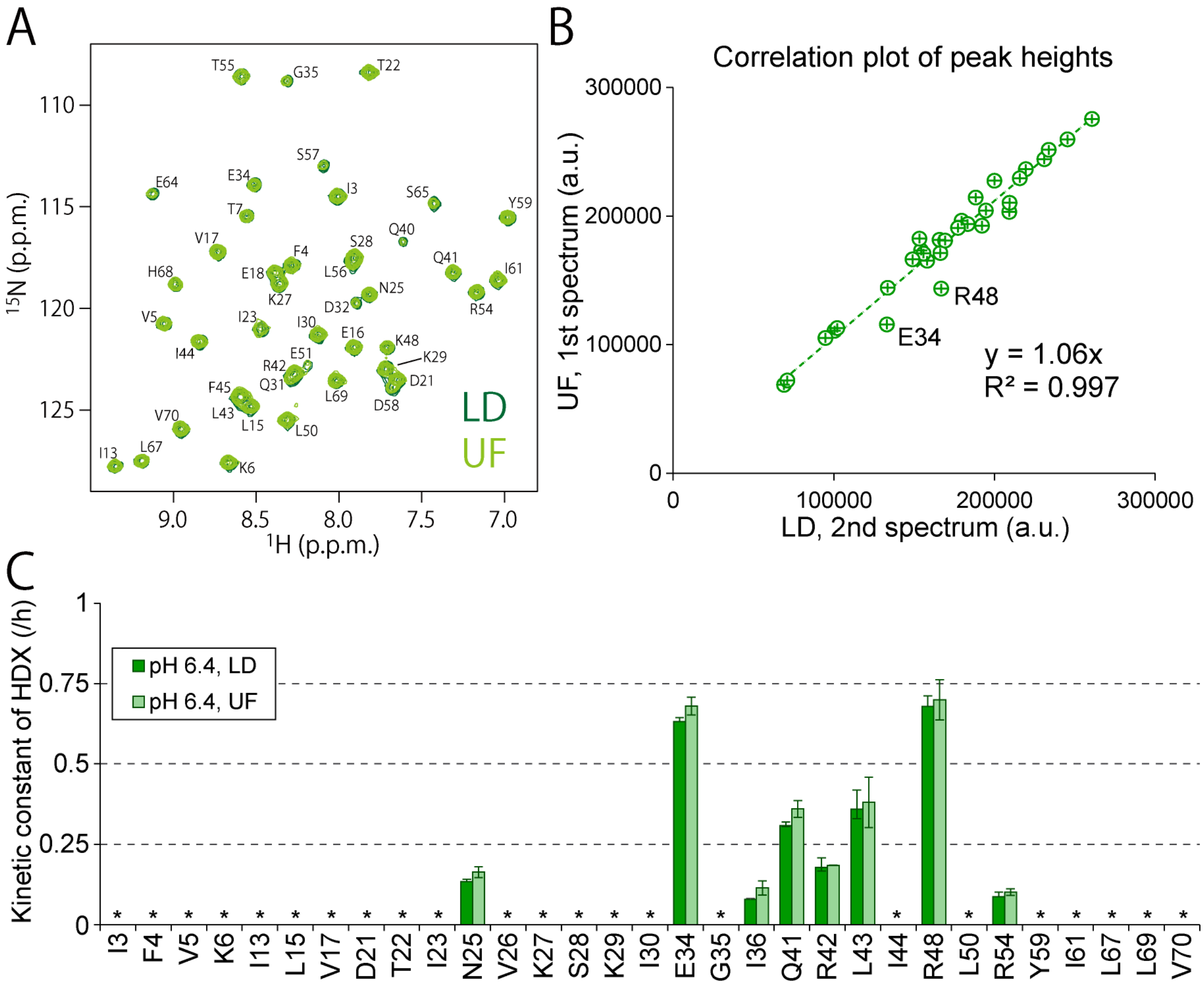

Supplementary Fig. S3. HDX profiles of Ub prepared by lyophilization-dissolution (LD) and buffer exchange by ultrafiltration (UF). (**A**) Overlaid amide ^{1}H-^{15}N SOFAST-HMQC spectra of Ub prepared by LD (deep green) and UF (light green), both of which were measured at 48 min (LD) and 51 min (UF) after exchanged into D_2O. NMR experiments were performed at 277 K in a 700 MHz magnet. (**B**) Correlation plot of peak heights between spectra shown in (**A**). Two remarkable outliers from the linear fit, E34 and R48, would reflect in the slight difference of delays before measurement (longer in the UF experiment by 3 min), as these two residues have the largest exchange rates as shown in (**C**). Error bars are based on noise levels. (**C**) HDX rate constants determined from LD and UF data. The mean values of duplicate experiments ± the differences are shown. HDX of the residues labeled with asterisks was too slow to determine the rate constant from the experimental condition.

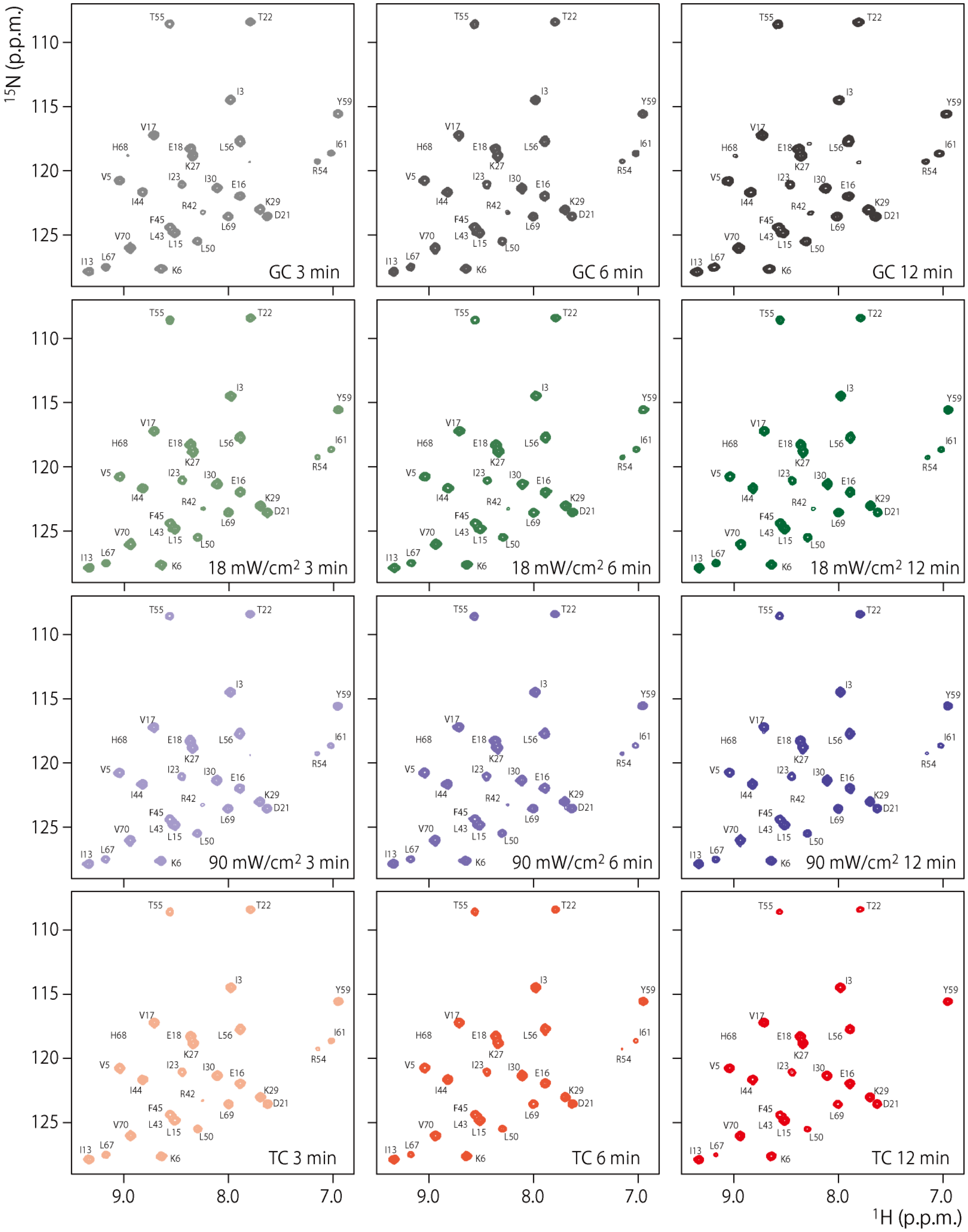

Supplementary Fig. S4. Amide ^{1}H-^{15}N SOFAST-HMQC spectra of THz-HDX samples.

A

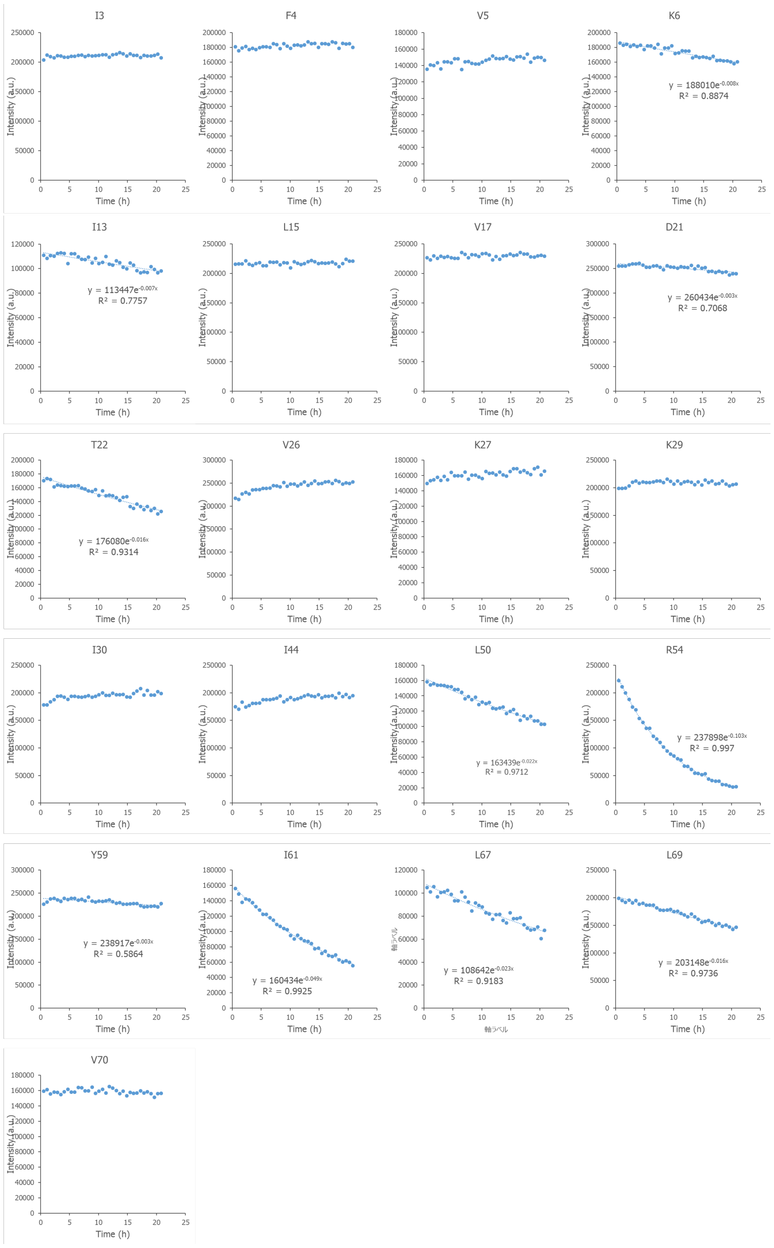
I3
F4
V5
K6
y = 188010e-0.008x
R² = 0.8874
I13
y = 113447e-0.007x
R² = 0.7757
L15
V17
D21
y = 260434e-0.003x
R² = 0.7068
T22
y = 176080e-0.016x
R² = 0.9314
V26
K27
K29
I30
I44
L50
y = 163439e-0.022x
R² = 0.9712
R54
y = 237898e-0.103x
R² = 0.997
Y59
y = 238917e-0.003x
R² = 0.5864
I61
y = 160434e-0.049x
R² = 0.9925
L67
y = 108642e-0.023x
R² = 0.9183
L69
y = 203148e-0.018x
R² = 0.9736
V70
Intensity (a.u.)
Time (h)

B

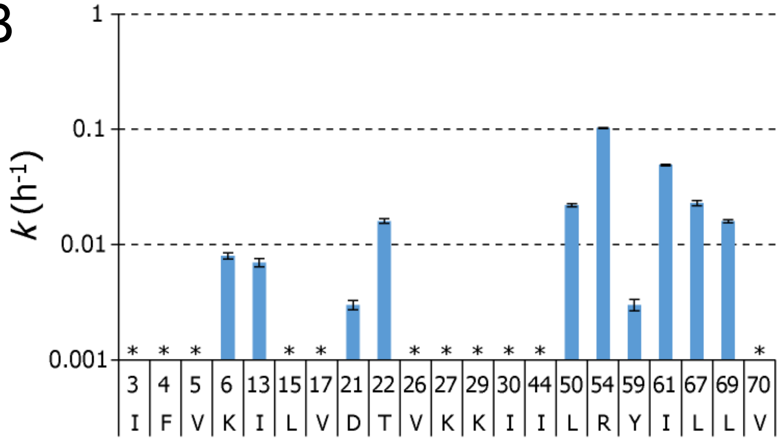
1
0.1
0.01
0.001
k (h-1)
3 4 5 6 13 15 17 21 22 26 27 29 30 44 50 54 59 61 67 69 70
I F V K I L V D T V K K I I L R Y I L L V

Supplementary Fig. S5. (**A**) Real-time NMR measurement of HDX in a GC sample. HDX profiles of the selected 21 amino acid residues were measured by NMR experiments just after dissolution of lyophilized Ub in D_2O. We used the same sample conditions, preparation procedure, and NMR experimental conditions as those used in the THz-HDX experiment, except that the NMR experiment was initiated 13 min after the dissolution and that spectra were acquired by Bruker Avance 700 Spectrometer equipped with a TXI triple resonance probe in 35 min 51 s per spectrum with 24 scans. V5, V26, K27, K29, I30, and I44 showed more than 3% increase in signal intensity. This might be due to decrease in transverse relaxation rates upon HDX of nearby labile proton sites. (**B**) The residue-specific rate constant of HDX, k, was obtained by fitting the data to an exponential decay equation $I = I_0 exp(-kt)$, where I_0 stands for the initial signal intensity without HDX. The residues were classified into three groups with the different rate constant k: I3, F4, V5, L15, V17, V26, K27, K29, I30, I44, and V70 have $k \approx 0$; T22, L50, L67, and L69 have $k\sim 0.02\ h^{-1}$; R54 and I61 have $k \sim 0.1\ h^{-1}$. Thus, in the first group with the smallest k, only less than 3% of signal intensity was reduced over the measurement time of ca. 20 h. Owing to the relatively large k, the THz/heat-induced effects on HDX were underestimated in several residues of the remaining groups (see Supplementary Fig. S7). Error bars correspond to errors of curve fitting by the exponential decay. HDX of the residues labeled with asterisks was too slow to determine the exchange rate under the experimental condition.

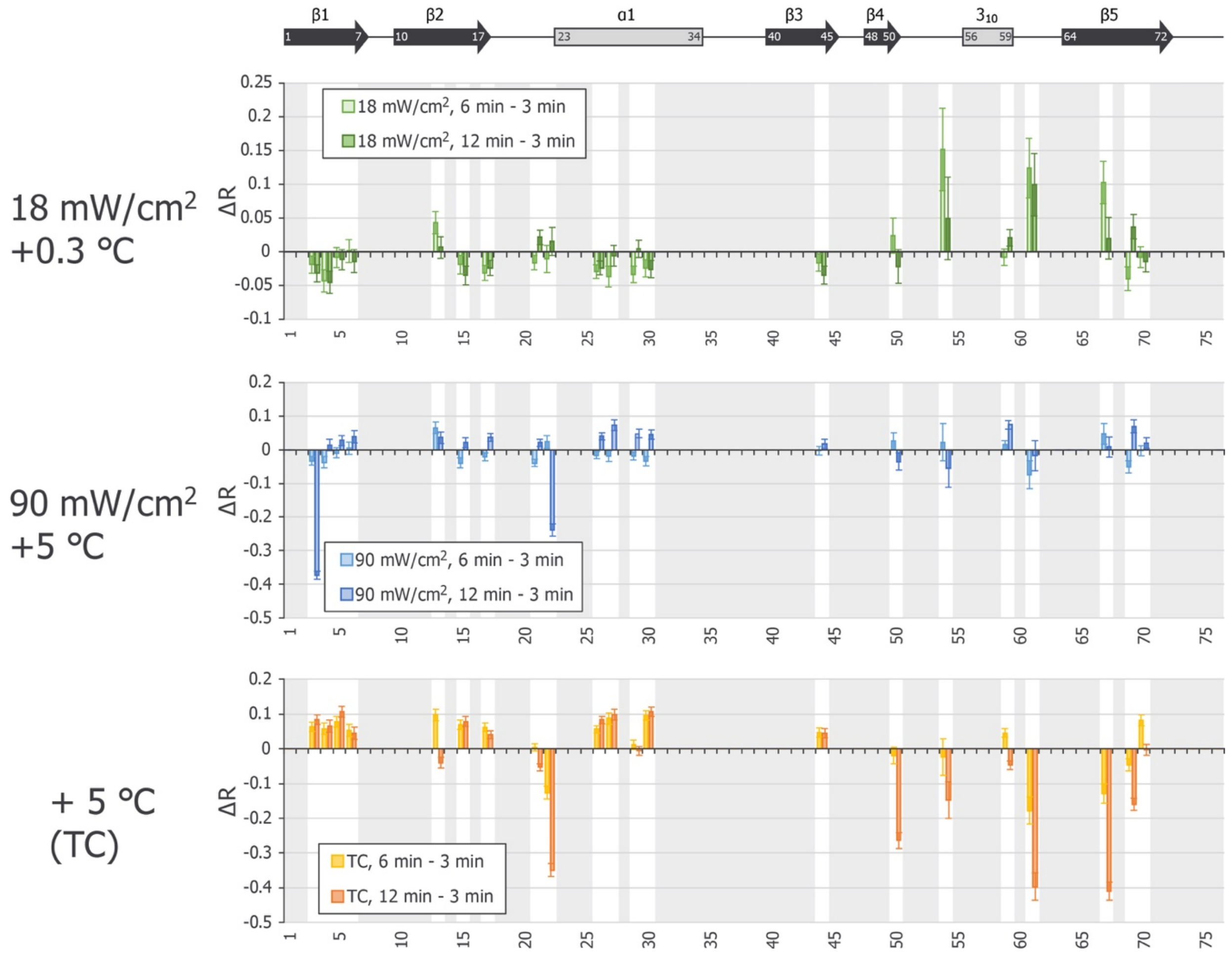

Supplementary Fig. S6. Effect of THz irradiation at low (top) or high (center) power density and the effect of temperature increase (bottom) on HDX changes through all amino acid residues of Ub. The secondary structure of Ub is also shown at the top of the graphs. TC represents the temperature control experiment. The magnitude of the effect (y-axis) is defined as ΔR, where R is the signal intensity ratio of each measurement to GC's measurement ($I/I_{(GC)}$). GC represents the general control experiment. ΔR is a difference in R at 6 or 12 min from that at 3 min. The residues excluded from the analysis (see Materials and Methods for the details) are indicated by gray color.

A

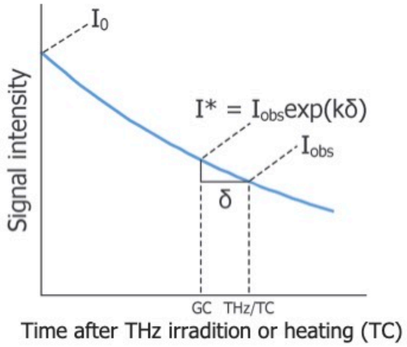

I0
I* = Iobsexp(kδ)
Iobs
δ
Signal intensity
GC
THz/TC
Time after THz irradition or heating (TC)

B

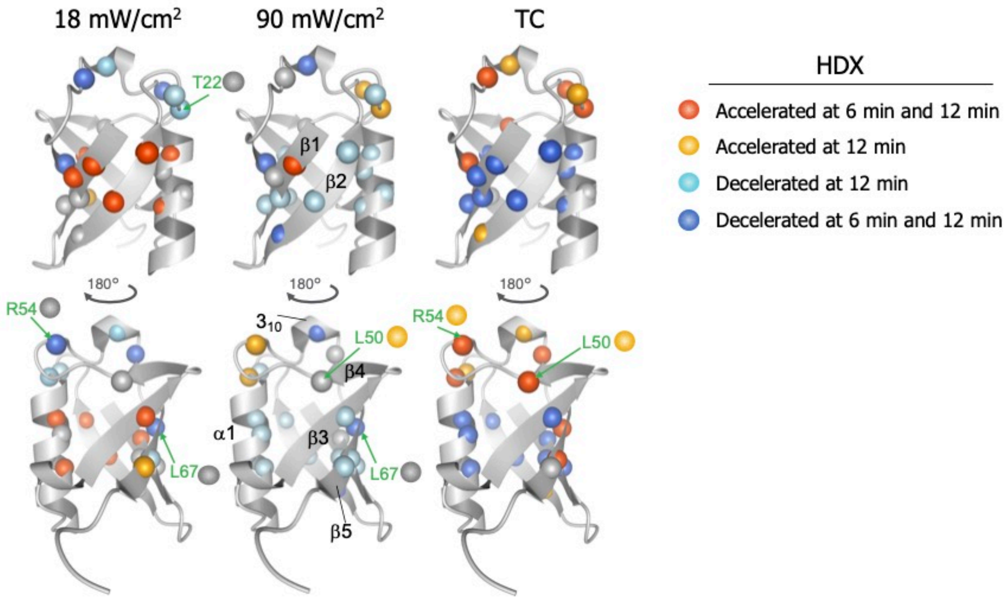

18 mW/cm2
90 mW/cm2
TC
HDX
Accelerated at 6 min and 12 min
Accelerated at 12 min
Decelerated at 12 min
Decelerated at 6 min and 12 min
T22
β1
β2
180°
R54
3₁₀
L50
β4
α1
β3
L67
β5

C

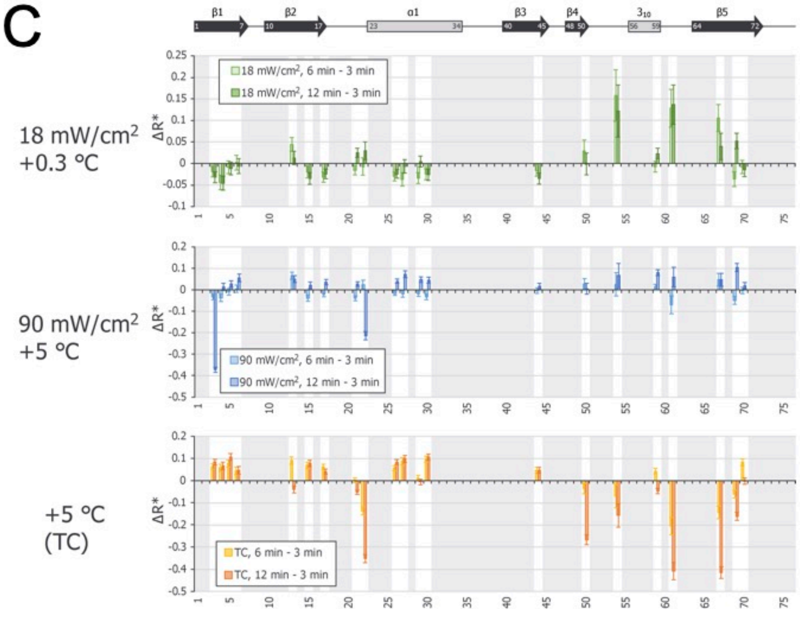

β1
β2
α1
β3
β4
3₁₀
β5
18 mW/cm2
+0.3 °C
18 mW/cm2, 6 min - 3 min
18 mW/cm2, 12 min - 3 min
90 mW/cm2
+5 °C
90 mW/cm2, 6 min - 3 min
90 mW/cm2, 12 min - 3 min
+5 °C
(TC)
TC, 6 min - 3 min
TC, 12 min - 3 min
ΔR*

Supplementary Fig. S7. Effect of sub-THz irradiation at low (left) and high (middle) power density and temperature increase (right, TC) on the amid proton exchange of Ub, in which the difference in the time intervals between the sample correction and NMR measurement (i.e. the interval between sub-THz/heat perturbations and NMR probing) were corrected. (**A**) The signal intensities of the sub-THz-irradiated and TC samples were corrected to have the perturbing and probing intervals equal to those of the GC samples. The corrected intensity I^* was estimated by an equation $I^* = I_{obs} exp(k\delta)$, where I_{obs} is the observed intensity, δ is the interval difference (Supplementary Fig. S2), and k is the residue-specific rate constant of HDX (Supplementary Fig. S5B). The corrected ratio difference ΔR^* was determined using I^*, according to equations (1) and (2) (see Materials and Methods). The ΔR^* profiles in the selected 21 residues were then mapped onto the tertiary (**B**) and secondary (**C**) structures of Ub, in the same manners as shown Fig. 2 and Supplementary Fig. S6, respectively. In the panel B, the difference between ΔR and ΔR^* profiles are indicated with arrows, the residue numbers, and the labels showing acceleration, deceleration, and no significant change in Fig. 2. As shown in the panel B, the correction of the signal intensity to produce the ΔR^* profiles successfully uncovered the sub-THz wave- or heat-induced changes in several additional residues (T22, R54, and L67 in 18 mW/cm^2, L50 and L67 in 90 mW/cm^2, and L50 and R54 in TC) that were not able to be detected in the uncorrected ΔR profiles, therefore allowed us to more prominently capture the opposite effect observed between the sub-THz irradiation and heating.

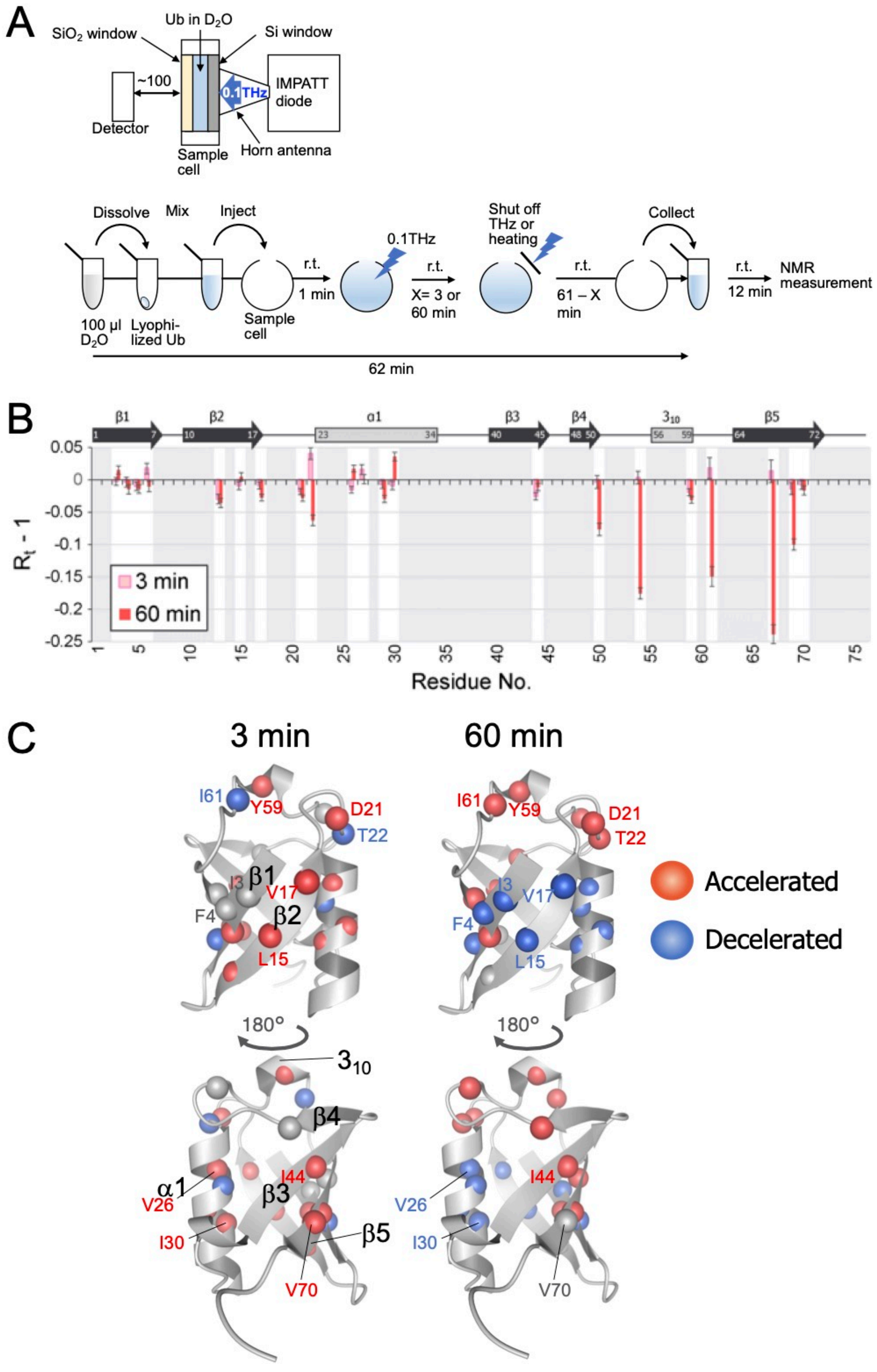
A
Ub in D_2O
SiO_2 window
Si window
~100
0.1THz
IMPATT diode
Detector
Sample cell
Horn antenna
Dissolve
Mix
Inject
Shut off THz or heating
Collect
0.1THz
r.t.
1 min
X= 3 or 60 min
61 – X min
12 min
NMR measurement
100 µl D_2O
Lyophi-lized Ub
Sample cell
62 min
B
β1
β2
α1
β3
β4
3_{10}
β5
R_t - 1
3 min
60 min
Residue No.
C
3 min
60 min
I61
Y59
D21
T22
I3
β1
V17
F4
β2
L15
180°
3_{10}
β4
α1
V26
I30
β3
I44
β5
V70
Accelerated
Decelerated

Supplementary Fig. S8. THz-HDX without a long interval between sub-THz perturbation and NMR probing**.** (**A**) Sub-THz irradiation to Ub solution using IMPATT diode-based light source. Details of the light source are described in Supplementary Methods. The length unit is shown in millimeter. Sample cell and detector are the same as those shown in Fig. 1A. The experimental procedure of the THz-HDX using the IMPATT diode device is schematically shown on the bottom (see the legend of Fig. 1C for more details). (**B**) Effect of sub-THz irradiation for 3 min (pink) or 60 min (red) on HDX through all amino acid residues of Ub. The secondary structure of Ub is also shown at the top of the graphs. The residues excluded from the analysis are indicated by gray color. The magnitude of the effect (y-axis) is defined as $R_t - 1$, where R_t is the signal intensity ratio of each measurement to GC's measurement ($I_{(THz)}/I_{(GC)}$), as defined by Eq. (1). GC represents the general control experiment. For the analysis of HDX data, we used $R_t - 1$, instead of ΔR (see Eq. (2)) used for analyzing the klystron data in Fig. 2 and Supplementary Figs. S5 and S7. This is because temperature elevation in 3 min was as small as 0.5 °C in the IMPATT diode-based experiment, and thus $R_{3\ min}$ data per se would mainly reflect the nonthermal effect as observed in the klystron-based irradiation at 18 mW/cm^2. In contrast, the irradiation for 60 min resulted in accumulative temperature elevation of 2°C, which supposedly rendered the 60 min data dominated by the thermal effect, as indicated in Supplementary Fig. S9. (**C**) The tertiary Ub structures with 180° rotation are shown (PDB accession code: 1UBQ). Amide nitrogen atoms of the analyzed residues are shown. Amino acid residues were mapped in the Ub structure when the HDX of the main chain amide groups was accelerated or decelerated. The acceleration or deceleration of HDX in each residue was defined using the signal intensity ratio of each measurement to GC's measurement ($I/I_{(GC)}$; see Materials and Methods for details). When HDX was accelerated (i.e., $0 > -Error(R_t) > R_t - 1$) or decelerated (i.e., $R_t - 1 > Error(R_t) > 0$), following sub-THz radiation above the measurement error range, the corresponding residue was colored red or blue, respectively. $Error(R_t)$ values are derived from signal-to-noise ratio, following Eq. (6).

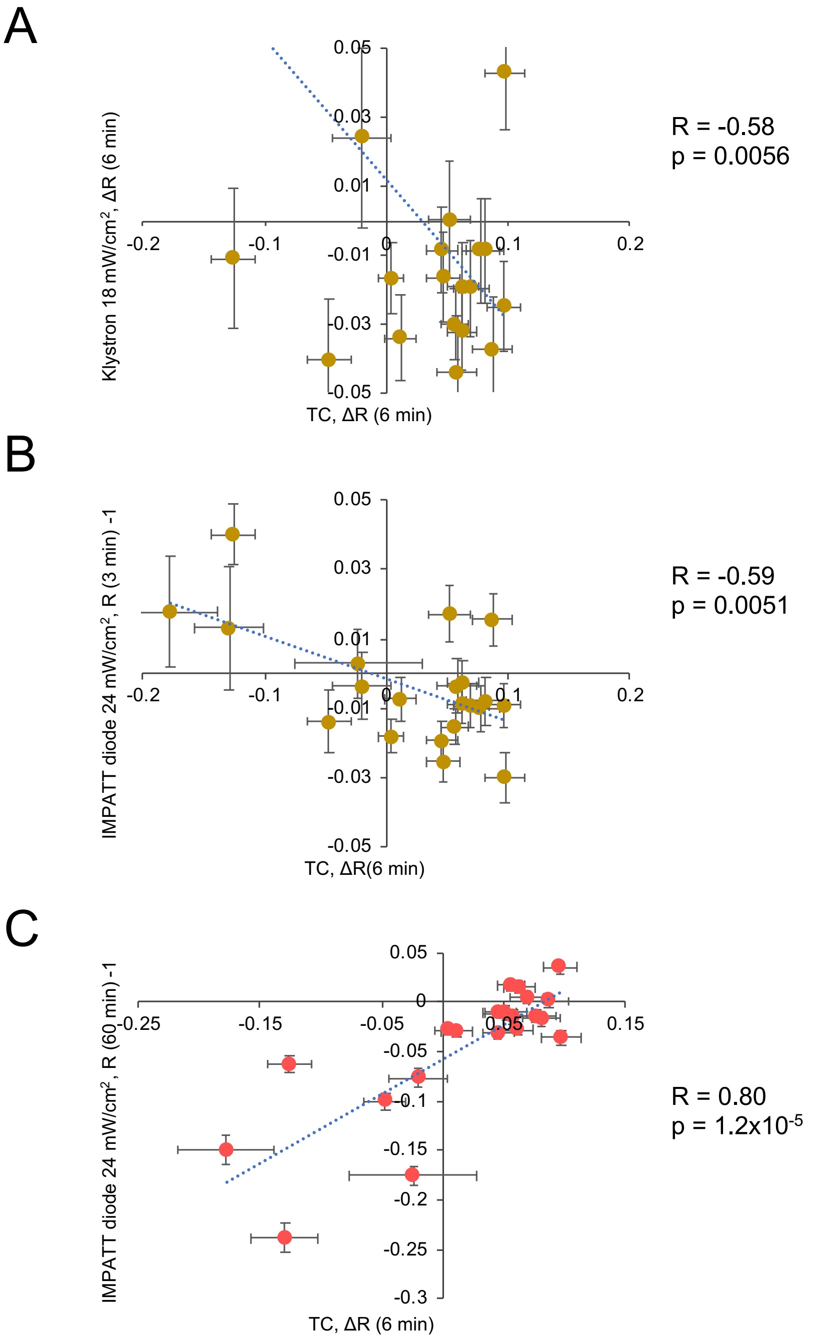

Supplementary Fig. S9. Correlation relationships of ΔR (or R_t -1, see Materials and Methods) between (**A**) klystron-based sub-THz irradiation at 18 mW/cm^2 for 6 min and TC for 6 min, (**B**) IMPATT diode-based sub-THz irradiation at 24 mW/cm^2 for 3 min and TC for 6 min, and (**C**) IMPATT diode-based sub-THz irradiation at 24 mW/cm^2 for 60 min and TC for 6 min. Error bars represent errors calculated with Eq. 5 (for the experiments with klystron device and TC) and with Eq. 6 (for the experiment with IMPATT diode device). Pearson correlation coefficient R between the two variables and the p-value are shown in each graph.

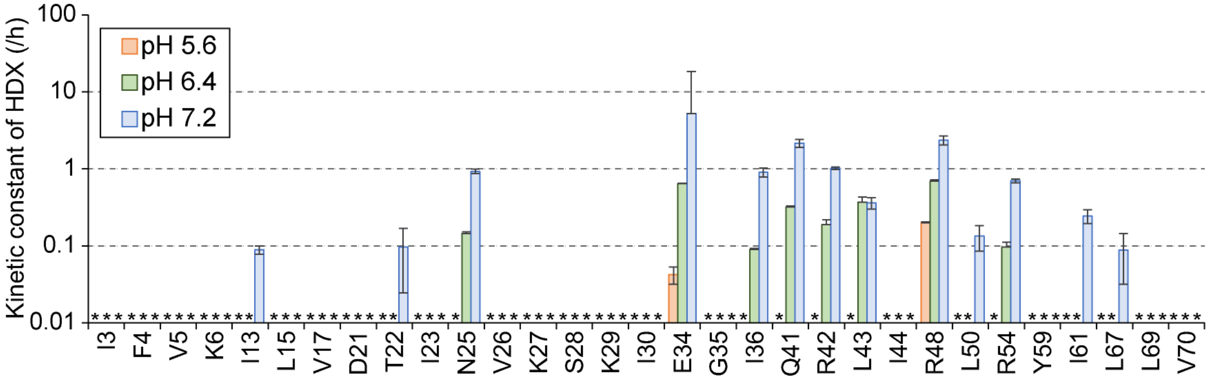

Supplementary Fig. S10. Rate constants for HDX under different pH conditions. The mean values of duplicate experiments ± the differences are shown. Experiments were performed at 277 K in a 700 MHz magnet. HDX of the residues labeled with asterisks was too slow to determine the rate constant under the experimental condition. Among 13 residues, whose rate constants were determined at pH 7.2, rate constants of 5 and 11 residues could not be determined at pH 6.4 and 5.6, respectively, due to the decelerated HDX.
